# Multi-Target Virtual Screening of Natural Products Against Virulence-Associated Proteins of *Pseudomonas aeruginosa* PAO1 for Antivirulence Candidate Prioritization

**DOI:** 10.3390/ph19101537

**Published:** 2026-09-28

**Authors:** Wenjie Chen, Yu Zhang, Jia Zeng, Yuan Wang, Bo Cheng

**Affiliations:** 1 School of Environmental Ecology and Biological Engineering, Wuhan Institute of Technology, Wuhan 430205, China; witwenjiechen@163.com (W.C.); 18792793843@163.com (Y.Z.); 15587593301@163.com (J.Z.); 18972038323@163.com (Y.W.); 2 Hubei Key Laboratory of Microbial Transformation and Regulation of Biogenic Elements in the Middle Reaches of the Yangtze River, School of Environmental Ecology and Biological Engineering, Wuhan Institute of Technology, Wuhan 430205, China

**Keywords:** *Pseudomonas aeruginosa*, antivirulence candidate prioritization, natural products, multi-target virtual screening, control-set sensitivity, molecular dynamics

## Abstract

**Background/Objectives**: Natural products offer chemically diverse starting points for antivirulence discovery. We aimed to prioritize candidates against ten *Pseudomonas aeruginosa* PAO1 virulence-associated proteins while accounting for differences in structural support and compound-access requirements. **Methods**: We screened 391,658 COCONUT-derived ligand entries using staged AutoDock Vina docking, followed by GNINA rescoring and within-target Binding–Access–Exposure–Liability (BAEL) prioritization. Scaffold profiling, ranking sensitivity analyses, and triplicate 100 ns molecular dynamics simulations of 30 selected complexes characterized chemical diversity, selection consistency, and trajectory behavior. A separate retrospective LasB analysis examined sensitivity to comparison design. No prospective biochemical or cellular validation was performed. **Results**: The workflow yielded 189 candidate–target associations involving 182 unique natural products across six functional classes. The 181 scaffold-bearing compounds represented 176 Bemis–Murcko families. BAEL retained 179 of 200 Binding-only selections, with property-informed substitutions concentrated near the selection cutoffs. Top-20 selection overlap remained high under prespecified 25% perturbations of the Access, Exposure, and Liability rules (Jaccard similarity ≥ 0.905). Of 90 trajectories, 82 were classified as pocket-proximal under the modeled conditions. In the LasB analysis, areas under the receiver operating characteristic curve were 0.700/0.740 for Vina/GNINA with surrogate controls and 0.480/0.480 in the attempted property-matching sensitivity set, which showed residual property imbalance and differed in annotated-parent coverage and weighting. **Conclusions**: This study connects large-scale natural-product screening with target-specific experimental planning. Binding and antivirulence activity remain to be established, but the annotated candidates provide a chemically diverse starting point for focused biochemical and cellular evaluation.

## 1. Introduction

*Pseudomonas aeruginosa* PAO1 is a widely used reference strain for studies of opportunistic infection and antivirulence strategies [1,2,3]. Antivirulence strategies aim to attenuate pathogenic functions without using bacterial growth inhibition as the sole endpoint [4,5,6]. The ten proteins examined here represent six virulence-related functions: extracellular proteolysis, type III secretion, toxin activity, type IV pilus function, motility–biofilm regulation, and alginate biosynthesis. LasB, LasA, and AprA are extracellular proteases [7,8,9]. ExoT and ExoY are type III secretion effectors [10,11,12,13], whereas exotoxin A (ToxA) inhibits host protein synthesis by ADP-ribosylating eukaryotic elongation factor 2 [14]. PilT and PilM contribute to type IV pilus motility and assembly [15,16,17]. FleQ regulates motile and biofilm-associated states [18], and AlgD contributes to alginate biosynthesis [19]. The panel was chosen to sample extracellular damage, host-directed toxin activity, and bacterial functions involved in motility, attachment, and matrix production.

Prior inhibitor studies provide pharmacological support for targeting LasB [20,21,22]. For the other panel members, genetic, functional, or biochemical evidence supports their virulence-related roles [8,9,10,11,12,13,14,15,16,17,18,19], but this biological evidence is distinct from the structural support for a particular docking setup. *P. aeruginosa* computational studies have used machine-learning target identification for antibiofilm molecules [23] or single-target docking, molecular dynamics (MD), and free-energy analysis for MvfR [24]. Natural-product virtual screening against LasR has combined docking with biochemical or biophysical evaluation [25]. A recent multitarget study evaluated a defined panel of natural bioactives across several *P. aeruginosa* virulence-related proteins, together with experimental phenotypes [26]. These designs address target-focused discovery, phenotypic target identification, or defined multitarget compound panels (Table S11). Multiparameter optimization can make property trade-offs explicit, but its interpretation depends on project objectives and component weights [27]. PilT, PilM, and AlgD remain exploratory structural configurations here despite their documented biological functions. Natural-product collections offer broad chemical coverage [28,29]; physicochemical profiling and structural-alert screening provide complementary descriptions of compound properties and liabilities [30,31,32,33,34,35].

The functional diversity of the panel creates distinct compound-access and assay requirements. Extracellular proteases can be tested directly in biochemical assays, whereas intracellular bacterial targets are constrained by membrane permeability and efflux [36,37,38,39,40,41,42,43]. Host-delivered effectors and ToxA require cell-compatible systems that connect target-proximal activity to host-cell phenotypes. A uniform score-only ranking does not account for these target-specific assay and access constraints.

We therefore screened a COCONUT-derived natural-product library against ten PAO1 virulence-associated proteins. We hypothesized that adding candidate-level Access compatibility, Exposure-related physicochemical proxies, and Liability to docking-derived Binding evidence would preserve the primary docking signal while making limited changes for assay planning. We evaluated this hypothesis within each target using the study-specific, weighted-sum Binding–Access–Exposure–Liability (BAEL) prioritization rule [44]. We then characterized chemical diversity, medicinal-chemistry risk, and representative trajectory behavior to define target-specific assay priorities. Because docking-score interpretation depends on receptor state, ligand representation, scoring assumptions, and benchmark construction [45,46,47,48,49,50,51], we conducted a separate LasB retrospective analysis to examine the sensitivity of apparent score discrimination to control-set construction. This retrospective analysis did not influence candidate selection.

## 2. Results

### 2.1. Multi-Target Screening Prioritizes 182 Natural-Product Candidates Representing Six Virulence-Function Classes

The screen yielded 391,658 ligand entries in Protein Data Bank format with partial charges and atom types (PDBQT), of which 391,544 received Vina scores for all ten targets. Within each target, Vina ranking retained 3916 complete-profile entries before validity filtering. Pocket-geometry assessment of the top 250 records supplied up to 150 records for three additional docking runs. The consistency and pose-geometry composite then selected 50 review records per target (500 in total; Supplementary Table S13). A five-seed Vina assessment was applied to the 50 review records per target and defined a 20-record repeat-docking support subset, while GNINA rescoring was performed for all 500 review records. BAEL ranked all 50 review records within each target. The top 20 were retained, yielding 200 screening records; the remaining 30 per target were assigned ranks 21–50. This fixed quota supports target coverage, not relative enrichment or hit-rate comparisons between targets. Grouping screening records by target and source-parent identifier yielded 189 candidate–target associations; counting source-parent identifiers across these associations yielded 182 unique compounds. Of these, 175 compounds were associated with one target and seven with two, covering all six virulence-function classes. Each association represents a separate target-specific experimental hypothesis. Analysis levels and retained counts are summarized in Table 1.

Library preparation started from 715,818 raw COCONUT records and yielded 398,520 standardized simplified molecular-input line-entry system (SMILES) records, of which 391,829 entered final PDBQT preparation (Figure 1A). No scaffold or parent-compound cap was applied during within-target selection.

The target panel linked each biological context to an initial experimental route (Table 2). LasB, AprA, LasA, and ToxA were assigned to the Relaxed Access class; ExoT and ExoY to the Intermediate class; and FleQ, PilT, PilM, and AlgD to the Strict class. These classes summarize assay access and exposure requirements and provide assay-planning context.

Seven compounds were each associated with two targets; their complete assignments are listed in Supplementary Table S9. For example, CNP0584125 was retained independently for FleQ and LasB.

### 2.2. BAEL Preserves Binding-Led Prioritization While Adjusting Selected Boundary Cases

BAEL combined four components into a within-target prioritization score: docking-derived Binding evidence, candidate-level Access compatibility, physicochemical Exposure proxies, and encoded Liability. The candidate-level Access component was calculated from a target-class baseline and compound properties and is distinct from the target-level Access class used to organize assay routes. All comparisons were performed within targets.

Compared with Binding-only ranking, BAEL had a median top-20 Jaccard similarity of 0.818 and shared 179 of 200 memberships. The other 21 memberships reflected target-specific substitutions at or just below the Binding-defined top-20 cutoff.

Of the 21 BAEL-versus-Binding substitutions, nine incurred a normalized Binding loss of no more than 0.05. Seven of these nine were non-inferior in Access, Exposure, and Liability and improved in at least one of the three components. Across eight prespecified 25% A/E/L rule-strength perturbations and ten targets, all 80 target-scenario comparisons retained top-20 Jaccard similarities of at least 0.905. Across the 30 target-by-concentration summaries from the Dirichlet weight analysis, target-wise median top-20 Jaccard values ranged from 0.818 to 1.000, median Spearman correlations from 0.968 to 0.999, and stable-core sizes from 15 to 20; alternative Binding inputs produced larger changes.

At the LasB top-20 boundary, CNP0525501 moved from Binding rank 21 to BAEL rank 19 and replaced CNP0216584, which moved from Binding rank 20 to BAEL rank 21. The entrant incurred a normalized B-score loss of 0.00112, while Exposure and Liability increased by 0.10 and 0.07, respectively, with Access unchanged. This illustrates a boundary trade-off rather than a reversal of the Binding signal. PilT/CNP0432643 and AlgD/CNP0433293 likewise changed within-target order from ranks 8 to 3 and 5 to 2, respectively. Full sensitivity results are reported in Supplementary Tables S6–S8 and Figures S1–S3.

### 2.3. Retained Candidates Span Diverse Scaffolds and Medicinal-Chemistry Profiles

Among the 199 structurally evaluable screening records, 151, 45, and three had zero, one, and two Lipinski violations, respectively; 79 had synthetic-accessibility scores of no more than 4.0. Figure 2B shows calculated log octanol–water partition coefficient (cLogP)–topological polar surface area (TPSA) space for these records, of which 147 were predicted to have high gastrointestinal absorption, and 196 were predicted not to cross the blood–brain barrier. Pan-assay interference compound (PAINS) annotations comprised 185 records with no alerts, 13 with one, and one with multiple alerts; 101 records had neither PAINS nor Brenk alerts. These annotations are predictive proxies rather than measured exposure or toxicity. LasB/CNP0267268, for example, had no Lipinski violation and a synthetic-accessibility score of 3.21, but its PAINS alerts and high post hoc risk place aggregation, metal-chelation, and readout-interference controls before mechanistic interpretation.

Structure-dependent analyses comprised 199 screening records and 181 scaffold-bearing unique compounds. The 181 compounds with computable non-isomeric Bemis–Murcko scaffolds formed 176 families: 171 singleton families and five doubleton families. The predominance of singleton families broadened chemical-space coverage but left only five two-member families for immediate series-level comparison.

### 2.4. Representative Candidates, Trajectory Behavior, and Testing Priorities

The three highest-ranked BAEL associations per target yielded 30 complexes and 90 100 ns trajectory replicates. Pocket-proximal required at least 80% common-pocket or pocket-core occupancy; surface-associated required below 20% common-pocket and at least 80% other-surface occupancy; sustained dissociation required loss of whole-protein contact, a minimum heavy-atom distance above 1.0 nm, and at least 10 ns of continuous separation. All other trajectories were pocket-shifted.

Counts were 82 pocket-proximal, six pocket-shifted, two surface-associated, and none dissociated; 24 of 30 complexes were concordant, and six were mixed. These proportions apply only to the prespecified three-per-target subset and describe trajectory behavior under the modeled conditions, not binding frequency, affinity, or activity. Supplementary Table S5 and Figure 3 provide replicate metrics and examples.

Classification criteria: pocket-proximal, ≥80% common-pocket or pocket-core occupancy; surface-associated, <20% common-pocket and ≥80% other-surface occupancy; sustained dissociation, loss of whole-protein contact with a minimum heavy-atom distance > 1.0 nm for ≥10 ns continuously; otherwise, pocket-shifted. Shared-pocket definitions and periodic-boundary-condition-aware processing are detailed in Section 4.6; replicate metrics are provided in Table S5. These classes describe trajectory geometry, not confirmed binding.

Table 3 links nine representative candidates to within-target ranks, trajectory classes, structural context, post hoc risk, and proposed first-line assays; PilT, PilM, and AlgD remain exploratory structural setups. Six representative pairs are illustrated in Figure 4.

CNP0407003 ranked first for ExoT under both BAEL and Binding-only ordering (BAEL score, 0.745486; Figure 5). All three trajectories were pocket-proximal, with mean positional ligand root-mean-square deviations (RMSDs) of 0.20, 0.22, and 0.47 nm; final-frame RMSDs of 0.17, 0.20, and 0.50 nm; and 100% common-pocket contact occupancy in each replicate.

LasB/CNP0267268 was classified as pocket-proximal in all three trajectories (Table 3).

### 2.5. Retrospective LasB Analysis Identifies Control-Set Sensitivity

This analysis was conducted independently of prospective screening and BAEL ranking to test whether apparent LasB score discrimination depended on control construction. We analyzed 195 parent compounds with LasB assay annotations as an assay-annotation set, retaining potency context at the assay level, and compared them with 893 evaluable surrogate controls. The annotated compounds were not treated as a uniformly active class, and the surrogate controls were unannotated comparators rather than experimentally confirmed LasB-negative compounds. Vina and GNINA affinity produced areas under the receiver operating characteristic curve (AUROCs) of 0.700 and 0.740, respectively. The controls had fewer aromatic rings than the annotated compounds (mean, 0.425 vs. 1.979; SMD, −1.802), indicating that the apparent separation was strongly associated with control-set composition.

Score-blind attempted property matching selected COCONUT controls before inspection of Vina or GNINA scores (Methods Section 4.7; Supplementary Section S1.6). At least one control was selected for 148 of 195 annotated parents (75.9%). Under parent-normalized weighting of these 148 parents and 705 complete-score controls, Vina and GNINA affinity each produced an AUROC of 0.480 (95% CIs, 0.411–0.536 and 0.410–0.533, respectively); both intervals included 0.5. Average precision was 0.469 and 0.466, with corresponding 95% CIs of 0.427–0.532 and 0.427–0.525. Both intervals included the weighted baseline of 0.5, so neither score showed enrichment above that baseline in this sensitivity set. The retained set did not meet the prespecified |SMD| ≤ 0.20 balance criterion: residual SMDs were −0.321 for heteroatom count, 0.272 for total rings, and −0.212 for hydrogen-bond donors.

The comparisons differed in annotated-parent coverage (195 vs. 148), control composition, and weighting (row-unweighted versus parent-normalized). The AUROC reduction from 0.700–0.740 with surrogate controls thus shows sensitivity to the joint comparison design. These simultaneous differences and the residual imbalance prevent attribution to property matching or any single cause. The estimates describe score behavior under these designs and cannot validate the screened candidates. Table 4 and Figure 6 summarize the results, with full details in Supplementary Figure S4 and Tables S12 and S19.

### 2.6. Exploratory LasB Structure–Label Analysis Shows Assay-Series-Specific Signal

A separate exploratory analysis used 192 records from two LasB assay families to assess the series-specific structure–label signal. Here, ECFP4 denotes an extended-connectivity fingerprint with diameter 4. ECFP4-plus-score and ECFP4 alone showed comparable performance, and transfer between assay families was weak. The analysis therefore identified predominantly local, series-specific structure–label relationships. This analysis was separate from candidate selection. Complete numerical results are provided in Supplementary Tables S14 and S15 and Figure S6.

## 3. Discussion

### 3.1. Candidate Landscape and Target-Specific Opportunities

The principal output is a target-stratified set of 189 candidate–target hypotheses involving 182 natural products across extracellular proteolysis, type III secretion, toxin activity, type IV pilus biology, motility–biofilm regulation, and alginate biosynthesis. This breadth supports comparison of assay requirements across distinct virulence functions, while the fixed target quotas do not support between-target enrichment claims. The predominance of singleton scaffolds broadens chemical-space coverage but limits immediate series-level structure–activity analysis. Initial experiments should therefore establish compound quality, target-proximal activity, and assay compatibility before extensive analog interpretation. If activity is reproduced, nearest-neighbor searching, scaffold hopping, or targeted derivatization can support later hit expansion.

The panel covers markedly different biological contexts. Extracellular proteases permit direct biochemical testing, whereas bacterial cytoplasmic targets require passage across both membranes and avoidance of efflux [52,53,54,55,56,57,58,59,60,61]. ExoT, ExoY, and ToxA require cell-compatible systems that connect target-proximal effects to host-cell phenotypes. Published studies support the roles of PilM/N/O/P in type IV pilus assembly, FleQ in c-di-GMP-responsive regulation, AlgD in alginate precursor synthesis, ExoY in cyclic-nucleotide production, and ExoT in Rho-family GAP activity and cell remodeling [12,13,17,18,19]. Structural support also varied: ExoT, FleQ, and LasA had low reported RMSDs in qualitative reference-ligand checks, whereas PilT, PilM, and AlgD had high reported RMSDs. The latter docking setups and their candidate binding geometries are therefore exploratory. These differences favor target-specific assay routes over a uniform whole-cell screen. For PilT, PilM, and AlgD, analog consistency should be evaluated only after target-proximal activity is established; it may guide follow-up prioritization but cannot by itself demonstrate target engagement.

### 3.2. Binding-Led Prioritization and Assay Design

BAEL retained 179 of 200 Binding-only memberships, with 21 substitutions. The LasB boundary example shows how Access, Exposure, and Liability annotations can distinguish assay-planning considerations for compounds with similar Binding scores. This decision-support role does not establish higher biological hit rates.

Alternative Binding inputs produced greater rank variation than the examined A/E/L perturbations. Docking scores depend on receptor conformation, pocket definition, ligand state, natural-product flexibility, and scoring-function assumptions [45,46,47,48,49,50,51]. Because Binding combines partially overlapping docking-derived channels, the sensitivity analyses characterize the prespecified rule rather than demonstrate feature independence or optimal weights.

Physicochemical and structural-alert annotations served a similar decision-support role. Conventional drug-likeness descriptors do not measure exposure in PAO1, and absorption, distribution, metabolism, and excretion (ADME) tools differ in endpoint definitions and applicability domains. These annotations identify solubility, aggregation, optical-interference, reactivity, permeability, efflux, and toxicity questions that should accompany target-specific assays.

### 3.3. Representative Trajectory Behavior and Structural Context

The MD analysis characterized selected starting poses across three 100 ns trajectory replicates. Pocket-proximal classifications identified poses that remained within the defined pocket region, whereas mixed classifications marked greater pose-level uncertainty. Because the 30 complexes were the three highest-ranked BAEL associations per target, the class proportions apply only to this selected subset. These descriptors and the post hoc quality-control summaries characterize trajectory behavior; they do not establish binding affinity, biological activity, or formal convergence. For PilT, PilM, and AlgD, these trajectories describe exploratory starting poses; pocket-proximal behavior does not resolve the high pose-recovery RMSDs or establish the correct binding mode.

CNP0407003 remains the primary ExoT candidate because its ranking was stable across the analyzed ordering scenarios. Its three trajectory replicates provide structural context for prioritizing the compound in initial ExoT testing. For LasB, AprA, and LasA, the trajectory analysis describes pocket proximity rather than zinc-coordination mechanisms. Follow-up evaluation should include metal-chelation controls and orthogonal assays.

### 3.4. LasB Scoring Across Comparison Designs and Assay Series

LasB score discrimination varied between the two comparison designs, with AUROCs of 0.700–0.740 for the surrogate-control comparison and 0.480 for both scores in the property-matching sensitivity analysis. The large aromatic-ring imbalance in the surrogate controls highlights the importance of examining control properties alongside score performance. Parent coverage, control composition, and weighting differed between analyses, and residual imbalance remained. The observed difference therefore reflects sensitivity to the comparison design rather than the isolated effect of property matching.

In the separate assay-series analysis, adding docking-derived scores did not clearly improve performance over ECFP4 alone, and transfer between assay families was weak. The observed structure–label relationships were mainly specific to the sampled assay series (Supplementary Tables S14 and S15).

### 3.5. Proposed Experimental Priorities

First, compound-quality controls would assess identity, purity, solubility, aggregation, optical interference, and target-relevant liabilities. LasB/CNP0267268 would additionally require metal-chelation controls, whereas any cell-based evaluation would require cytotoxicity and bacterial-growth controls.

Second, target-proximal assays should evaluate the proposed molecular function. CNP0407003 should first be tested in an ExoT GAP assay, CNP0178212 in a FleQ-centered regulatory assay, and CNP0267268 against purified LasB with LasA and AprA selectivity counterscreens. Candidates for PilT, PilM, AlgD, and ToxA require biochemical or biophysical formats appropriate to their target functions.

Third, compounds with reproducible target-proximal activity after interference controls should advance to cellular or virulence-phenotype assays. ExoT studies can use host-cell rounding with controls specific to the type III secretion system (T3SS), FleQ studies can measure motility and biofilm phenotypes, and extracellular-protease candidates can be evaluated in culture-supernatant assays. PilT, PilM, and AlgD remain exploratory priorities; negative cellular results should be considered together with bacterial permeability and efflux measurements.

### 3.6. Limitations

No prospective biochemical or cellular validation was performed in this study. All 189 candidate–target associations are computational hypotheses requiring experimental evaluation of activity, selectivity, exposure, target engagement, antivirulence effects, and growth-independent mechanisms. The LasB retrospective analyses are limited by the joint comparison design and residual property imbalance (Section 3.4). The separate structure–label analysis covered two assay-specific series and yielded predominantly series-specific signal.

The structural analyses reflect the receptor, ligand, and ion states represented in the docking and MD models. Docking used fixed receptor conformations and standardized prepared ligand states, so the results apply to those modeled structural states. Gasteiger partial charges were used for 29 simulated complexes and AM1-BCC for one, while metal-containing systems used standard AMBER ion parameters. Accordingly, the MD analyses characterize pocket proximity, pose drift, and surface relocation under the modeled conditions rather than quantitative binding affinity or coordination mechanisms.

ADME, toxicity, and structural-alert predictions served as proxy annotations for experimental planning. Access classes summarize target localization and assay context rather than experimentally measured compound accessibility. Because most scaffold families are represented by individual compounds, active candidates will require prospective hit expansion before series-level structure–activity relationships can be established. Finally, the fixed per-target selection design preserves coverage across all ten targets and supports coverage and prioritization summaries, not estimates of relative enrichment, hit rates, or binding affinity across targets.

## 4. Materials and Methods

### 4.1. Target Panel and Receptor Preparation

Ten virulence-associated proteins from *P. aeruginosa* PAO1 were selected using the Virulence Factor Database (VFDB), Victors, the Pseudomonas Genome Database/PseudoCAP, and published functional annotations [62,63,64]. The panel comprised LasB, LasA, and AprA; ExoT and ExoY; ToxA; PilT and PilM; FleQ; and AlgD. The selection rationale combined documented virulence-related functions with receptor models and defined docking regions available for this screen. The proposed first-line assays in Table 2 connect each target to a measurable functional readout. Structural availability supported computational tractability; it did not by itself establish ligandability or therapeutic validity. PilT, PilM, and AlgD were retained as exploratory structural setups to extend functional coverage; their high pose-recovery RMSDs limit confidence in the proposed binding geometries.

Receptor structures were prepared in PDB [65] and PDBQT formats using target-specific structural models and docking sites. ToxA (1AER), AlgD (1MFZ), and LasB (1EZM) retain their recorded screening-receptor labels. Supplementary Table S3 lists the prepared inputs, chain selection, coordinate coverage, retained ions, and docking-box definitions. Supplementary Section S1.1 distinguishes these labels from retrospective PDB correspondences and reference-ligand structure IDs. Receptor PDBQT files were prepared using mk_prepare_receptor in Meeko [66], and the same prepared receptor structure was used throughout docking for each target.

Mg^2+^ was retained for FleQ; Zn^2+^ and Ca^2+^ for AprA and LasB; and Zn^2+^ for LasA; PilT, PilM, AlgD, and ToxA were modeled in the apo state. ExoT, FleQ, and LasA used ligand-centered boxes. The FleQ/AGS and LasA/TLA structures corresponded to PDB entries 5EXS and 3IT7, respectively [9,67], and the F4W-associated ExoT configuration defined its ligand-centered docking region. ToxA, AprA, ExoY, and LasB used site- or metal-centered boxes, whereas PilT, AlgD, and PilM used predefined target-specific boxes. Exact box dimensions and receptor metadata are provided in Supplementary Table S3. ECFP4-constrained surrogate decoys accompanied the three reference-ligand pose-recovery checks [68,69].

### 4.2. Ligand Preparation, Docking, and Target-Wise Curation

The COCONUT-derived library initially contained 715,818 records associated with natural products. Open Babel 3.1.1 and RDKit 2024.03.5 were used for structure standardization, conversion, and descriptor calculation [70,71]. Standardization yielded 398,520 SMILES records; 391,829 entries entered final three-dimensional/PDBQT preparation, of which 391,658 were PDBQT-ready. Docking used the standardized prepared ligand states, with explicit source stereochemistry preserved where defined. Parent identifiers refer to source compounds, whereas prepared-state identifiers refer to structures used for docking.

Initial docking (Stage A). Docking was performed with AutoDock Vina 1.2.6 using the target-specific boxes listed in Supplementary Table S3 [45,46]. Initial docking used an exhaustiveness of 2, one output pose, and an energy range of 2 kcal/mol; 391,544 entries were scored for all ten targets.

Score-based selection and validity filtering (Stage B). For each target, the top 3916 records were retained, corresponding to 1% of the complete-profile library rounded up to the next integer. Records were ordered by ascending Vina score, and subsequent validity filters retained 3705–3915 records per target. Target-specific boundaries and retained counts are reported in Supplementary Table S13.

Pocket-geometry filtering (Stage C). Pocket-geometry filtering evaluated the 250 highest-ranked records per target. Contacts per ligand atom were used when density values were available; otherwise, absolute contact counts were used. Eligible poses were located inside the docking box, had a minimum receptor distance of no more than 3.0 Å, and met a threshold of at least 0.70 contacts per ligand atom or 15 absolute contacts. Records were ordered by Vina score, minimum distance, contact metric, center distance, and ligand identifier.

Three-run repeat docking (Stage D). Up to 150 records per target were retained for three additional Vina runs using an exhaustiveness of 16, up to ten poses, and an energy range of 5 kcal/mol.

Composite ranking (Stage E). The consistency assessment required three successful runs, a score standard deviation of no more than 0.25, an in-box pose, and complete metrics. The primary-to-mean difference was the absolute difference between the primary Vina score and the three-run mean, whereas the best-to-mean difference was the absolute difference between the best of the three scores and that mean. Both terms were direction-aligned before within-target winsorization at the 5th and 95th percentiles and min–max scaling. The composite weights were 0.20 each for minimum distance, the Stage C contact metric, and the three-run mean; 0.15 for score standard deviation; 0.10 for the primary-to-mean difference; and 0.05 each for center distance, the best-of-three score, and the best-to-mean difference. Records were ranked by the Stage E composite score, followed by best-of-three score, standard deviation, minimum distance, and ligand identifier. The 50 highest-ranked eligible records per target formed the review pool; they were not randomly sampled.

Five-run repeat docking (Stage F). Five-run repeat docking used nominal seeds 11, 22, 33, 44, and 55, an exhaustiveness of 64, up to 20 poses, and an energy range of 5 kcal/mol for all 50 Stage E records per target. The five-run mean, standard deviation, best score, Stage E score, and ligand identifier were used to define a 20-record repeat-docking support subset per target, with a score standard deviation threshold of 0.20.

GNINA rescoring (Stage G). GNINA v1.3.1 was used in score-only mode to rescore the model-1 poses of all 500 review records [47]. The repeat-docking summaries were retained as support features, whereas GNINA rescoring and BAEL ranking used the complete 50-record target pools. Records were ordered by CNN_VS, CNNaffinity, CNNscore, the Stage E score, and ligand identifier to obtain the GNINA ranking.

The 50 Stage E records per target formed the prespecified 500-record review pool. BAEL ranked all 50 records and retained the top 20 per target; the remaining 30 received ranks 21–50. This fixed per-target quota was used for coverage rather than estimation of between-target enrichment. No scaffold or parent-compound cap was applied. Screening records sharing a target and source-parent identifier were grouped into one association, represented by the record with the lowest within-target BAEL rank (Supplementary Table S18). This produced 189 associations; counting unique source-parent identifiers across targets yielded 182 compounds (Supplementary Table S1). The three highest-ranked BAEL associations per target were selected for MD, yielding 30 complexes. A compound retained for two targets was represented by two separate target-specific associations. The fixed three-per-target MD design ensured representation of all ten targets; trajectory-class proportions were therefore used descriptively and not to estimate hit rates or compare absolute binding strength across targets. Independent recalculation of the primary score and membership is documented in the Supplementary Methods.

### 4.3. Four-Component BAEL Prioritization Rule

Within each target, BAEL was implemented as a prespecified weighted-sum multicriteria prioritization rule [44]. The BAEL decision-support score was calculated as (*w_B_* × B) + (*w_A_* × A) + (*w_E_* × E) + (*w_L_* × L). Binding was a weighted sum of six components: the Stage E pose-geometry and repeat-docking composite (0.35), normalized Vina affinity (0.25), CNNscore (0.15), normalized CNN_VS (0.10), one minus the target-internal min–max-scaled GNINA cnnvariance field (0.10), and repeat-docking support (Stage F; 0.05). GNINA’s cnnvariance output was transformed into an internal consistency feature by target-wise normalization. Repeat-docking support was assigned as 1.0 when both repeat-docking rank and best-of-five score were present, 0.8 when one was present, and 0.6 when neither was present. All terms were direction-aligned so that larger values indicated greater support, and B was clipped to 0.05–1.00. The coefficients were prespecified operational weights for within-target prioritization.

The largest coefficient was assigned to the Stage E composite because it integrated pocket placement, local contact geometry, and consistency across repeated Vina runs. The normalized Vina term preserved the primary continuous affinity-ranking signal. GNINA-derived terms provided complementary rescoring information, with CNN_VS weighted below CNNscore because it partially incorporates CNNscore. Repeat-docking support was capped at 0.05. These channels were partially overlapping; their coefficients specified relative influence within the operational ranking and were not interpreted as independent probabilities or calibrated effect sizes.

The Access component started from a target-class baseline of 0.90, 0.80, or 0.72 for Relaxed, Intermediate, or Strict targets and applied candidate-specific deductions for molecular weight, TPSA, rotatable bonds, cLogP, and formal charge before clipping to 0.25–1.00. Exposure started from 0.88, 0.82, or 0.76 and applied class-specific deductions for cLogP, rotatable bonds, charge, and molecular weight before clipping to 0.20–1.00. Liability started at 1.00 and applied penalties of 0.15, 0.07, or 0.05 for severe, moderate, or other encoded alerts. An additional 0.10 penalty was applied when a DROP flag was accompanied by a documented exclusion reason. Molecular weight above 750 Da and TPSA above 200 Å^2^ each incurred a 0.05 penalty before clipping to 0.35–1.00. Records without an encoded alert received no alert penalty.

The class-specific weights (*w_B_*, *w_A_*, *w_E_*, *w_L_*) were (0.72, 0.08, 0.05, 0.15) for Relaxed targets, (0.60, 0.15, 0.10, 0.15) for Intermediate targets, and (0.50, 0.20, 0.20, 0.10) for Strict targets. The coefficients were prespecified operational weights. The target-level Access class defined the assay context, whereas the candidate-level Access component quantified compatibility within that context. BAEL supports within-target prioritization. Complete component definitions, default rules, and sensitivity settings are provided in Supplementary Table S16. Binding retained the largest class-specific weight because the ranking was anchored to within-target docking evidence. Strict targets assigned greater relative weight to Access and Exposure because candidates for bacterial intracellular targets must traverse both membranes and avoid efflux, whereas Relaxed targets permit more direct target-proximal assays. These weights were specified before the reported sensitivity analyses; they were neither fitted to activity data nor optimized retrospectively.

Sensitivity analyses used the primary top-20 selections as the fixed reference set within the prespecified 50-record target pools. They evaluated Binding-only and dimension-ablation orders, alternative Binding inputs, Access implementations, A/E/L strengths, Dirichlet weight draws, and within-target A/E/L permutations. Full methods and outputs are reported in the Supplementary Methods, Tables S6–S8, and Figures S1–S3. Access classes also informed assay routes outside the numerical score. Extracellular-protease candidates were assigned to enzyme or culture-supernatant assays; intracellular bacterial targets required permeability and efflux assessment; and type III secretion effectors and ToxA required host-cell-compatible confirmation where appropriate.

### 4.4. ADME, Structural-Alert, and Scaffold Analysis

SwissADME provided rule-of-five descriptors, gastrointestinal-absorption predictions, blood–brain barrier predictions, and synthetic-accessibility estimates. pkCSM provided hERG, Ames, and hepatotoxicity predictions, whereas ADMETlab 3.0 provided additional annotations [30,31,32,33]. We retained molecular weight, cLogP, hydrogen-bond counts, TPSA, rotatable bonds, synthetic accessibility, and selected risk flags. PAINS and Brenk alerts were recorded separately [34,35]. Additional ADMET predictions were reported separately to support experimental planning, whereas BAEL used the components defined in Section 4.3.

The post hoc ADME risk tier was assigned after BAEL ranking. High risk was assigned when L < 0.70, an encoded structural alert was present, or an external-toxicity flag was positive. In the absence of a high-risk flag, moderate risk was assigned for molecular weight above 600 Da, TPSA above 140 Å^2^, Crippen cLogP above 5.0, more than ten rotatable bonds, or an external-toxicity caution. Low risk indicated that none of these conditions were present. Structure-dependent analyses comprised 199 screening records. Supplementary Table S16 provides the combined external-toxicity rules.

Canonical non-isomeric Bemis–Murcko scaffolds were calculated with RDKit at the unique-parent level [71]. Structure-dependent scaffold analysis comprised 181 candidates. Families were defined by identical canonical scaffold SMILES. Supplementary Tables S2 and S4 provide record-level annotations and scaffold assignments.

### 4.5. Reference-Ligand and Pose Checks

Six reference-ligand checks had reported heavy-atom pose-recovery RMSDs (Table S3). Reported RMSDs of 2.0 Å or lower were interpreted as supportive qualitative pose recovery for ExoT, FleQ, and LasA (1.14, 0.79, and 0.62 Å, respectively); values above 2.0 Å were treated as non-supportive and exploratory for PilT, PilM, and AlgD (10.78, 10.44, and 9.35 Å, respectively). AprA, ExoY, ToxA, and LasB were interpreted from site-based or annotated structural setups rather than a reported reference-pose RMSD. Supplementary Table S3 reports the receptors, reference ligands, boxes, scores, and available RMSDs. These categories describe the available structural evidence. Up to 50 SELFIES-mutated decoys per ligand were selected within 15% molecular weight, 1.5 cLogP units, 15 Å^2^ TPSA, one hydrogen-bond donor or acceptor, two rotatable bonds, and identical formal charge; ECFP4 Tanimoto similarity was below 0.35 [68,69]. Reference ligands and decoys were docked with the same receptors, boxes, and parameters.

The ExoT/CNP0407003 structural visualization was generated from the 0 ns configuration of trajectory replicate 1, processed for periodic boundary conditions (PBC).

### 4.6. Molecular Dynamics and Trajectory Classification

Each of the 30 representative complexes was analyzed using three 100 ns trajectory replicates in GROMACS [72]. Proteins were parameterized with AMBER99SB-ILDN and solvated in TIP3P water [73,74], whereas ligand parameters used GAFF2 [75,76]. Partial charges were assigned with Gasteiger for 29 complexes [77] and AM1-BCC for ExoT/CNP0339020.0 [78]; each complex used the same assignment across its three trajectories. Topologies were generated with ACPYPE 2023.10.27 and Antechamber [79,80]. All simulated ligand topologies had net charges of approximately zero. Dodecahedral systems were neutralized and adjusted to approximately 0.15 M NaCl. Zn^2+^, Ca^2+^, and Mg^2+^ were represented using standard AMBER ion parameters. No specialized Zn-coordination model or additional coordination restraints were used. Per-complex ligand parameterization and simulation settings are provided in Supplementary Table S17.

After minimization, equilibration at constant number of particles, volume, and temperature (NVT) used a velocity-rescaling thermostat [81] at 300 K for 100 ps, and equilibration at constant number of particles, pressure, and temperature (NPT) used a Parrinello–Rahman barostat [82] at 1 bar for 500 ps. End-of-NPT dodecahedral box-vector lengths averaged 9.360 nm for a, b, and c (range, 6.845–11.636 nm); systems contained a mean of 18,771.4 TIP3P water molecules (range, 6719–34,262).

The three trajectory replicates for each complex shared one solvated coordinate set before NVT. Velocities were generated at the start of NVT, but the realized velocity-generation seeds were not recovered from the retained text records. The 90 NVT logs recorded 90 distinct stochastic V-rescale thermostat seeds, with three distinct values per complex. NPT and production did not regenerate velocities. Production trajectories lasted 100 ns with a 2 fs integration step, and coordinates were written every 10 ps. Particle-mesh Ewald electrostatics [83] and LINCS constraints [84] were used.

Post hoc descriptive quality control covered the existing protein-backbone RMSD series for all 90 production trajectories and potential-energy series for all 90 NVT and 90 NPT runs. The final 20% of recorded time was compared with the preceding 20% using late-window means, dispersion, linear slopes, and window-to-window changes (Supplementary Section S1.5 and its accompanying QC tables). No prespecified numerical stabilization or convergence threshold was recovered from the retained records; these summaries therefore describe trajectory behavior without establishing formal convergence [85]. Descriptor analysis sampled 101 frames at 1 ns intervals.

Before analysis, complete molecules were reconstructed, periodic jumps were removed, systems were centered, and coordinates were fitted to the protein backbone. For each complex, the shared pocket was defined from the common pre-MD system as protein residues within 0.50 nm of ligand heavy atoms; the same residue set was applied to all three trajectory replicates. The residue identities, initial distances, and 0.80/1.55 nm core/shell radii are provided in Supplementary Section S1.5 and its accompanying shared-pocket definition table. Positional ligand RMSD was calculated after protein-backbone fitting without ligand self-fitting. Periodic-boundary-condition-aware heavy-atom contacts used a cutoff of 0.45 nm.

Pocket-proximal trajectories had common-pocket or pocket-core occupancy of at least 80%. Surface-associated trajectories had common-pocket occupancy below 20% and other-surface occupancy of at least 80%. Sustained dissociation required loss of whole-protein contact, a minimum heavy-atom distance above 1.0 nm, and at least 10 ns of continuous separation on the 1 ns grid. Trajectories not meeting these criteria were classified as pocket-shifted. These categories describe trajectory geometry under the specified simulation conditions.

### 4.7. LasB Retrospective Control-Set Analyses

LasB *K_i_* and IC_50_ annotations were curated from ChEMBL and BindingDB [86,87]. Reconciled parent records retained assay type, units, species, and construct information where available, producing 195 assay-annotated parent compounds. The 195 parent compounds were analyzed as an assay-annotation set with assay-specific potency context.

The surrogate-control analysis and a score-blind attempted property-matching sensitivity analysis evaluated Vina and GNINA affinity against their respective controls. CNNscore and CNN_VS were additionally evaluated in the supplementary sensitivity analyses. Scores were direction-aligned so that larger values indicated more favorable rankings. Descriptor distributions, ECFP4 similarity, and grouped out-of-fold property models were used to assess control-set composition.

For the score-blind attempted property-matching sensitivity analysis, controls were selected deterministically from standardized PDBQT-ready COCONUT structures before any Vina or GNINA score was inspected. Database-annotated compounds, surrogate controls, main-study candidates, exact standardized duplicates, exact annotated-parent Murcko-scaffold overlaps, and structures with ECFP4 Tanimoto similarity of at least 0.35 to any annotated parent were excluded. Strict matching required exact formal charge and aromatic-ring count, together with prespecified tolerances for total rings, molecular weight, cLogP, TPSA, hydrogen-bond donors and acceptors, rotatable bonds, heavy atoms, aromatic-atom fraction, and fraction sp^3^. Post-selection balance was evaluated against a prespecified |SMD| ≤ 0.20 criterion. Prespecified relaxed tiers were applied only when fewer than three strict candidates were available. During initial global allocation, each source entry was used at most once, and no more than five controls were assigned per parent.

At least one control was selected for 148 of 195 annotated parents (75.9%), yielding 714 rows with 714 unique source IDs. Standardized-InChIKey deduplication then retained 710 unique structures; 705 had complete four-score profiles, and all 148 covered parents remained represented in the parent-normalized analysis with equal total weight per parent. The remaining 47 parents received no selected control under the combined fixed structure/similarity exclusions, descriptor tolerances, and source-entry nonreuse rule. The resulting set did not meet the prespecified balance criterion (maximum |SMD|, 0.321). Complete tolerances, tier sequences, and the coverage breakdown are reported in the Supplementary Methods.

### 4.8. Exploratory, Series-Specific LasB Structure–Label Analysis

Cross-fold domain overlap was assessed using exact Bemis–Murcko scaffolds [88]. Butina clustering defined structure groups [89], and nested grouped validation used scikit-learn [90]. The exploratory dataset comprised 192 experimental-source records: 160 *K_i_* and 32 IC_50_ records [91,92], including 65 positive-labeled and 127 negative-labeled records. Fifty-four negative-labeled *K_i_* records contributed categorical assay labels rather than numerical potency values. The model combined ECFP4 with seven docking-derived score channels; Access, Exposure, and Liability were excluded. Comparators included Vina, an independent supervised Binding baseline, and ECFP4-only logistic regression. Full model specifications, split definitions, software versions, and cross-assay validation settings are provided in the Supplementary Methods and Tables S14 and S15.

### 4.9. Statistical Analysis

Results were summarized as means, standard deviations or standard errors, counts, proportions, and ranking metrics, as appropriate. LasB AUROC, average precision, and enrichment metrics were calculated separately for the surrogate-control and attempted-matching sensitivity analyses. In the covered-parent analysis, the assay-annotated and control groups each had total weight 1, giving an average-precision reference baseline of 0.5; the controls associated with each parent shared an equal total weight (Supplementary Section S1.6). Confidence intervals used 5000 cluster-bootstrap resamples at the annotated-parent level. Paired differences were summarized with corresponding bootstrap intervals. Descriptive two-sided tail probabilities were calculated as twice the smaller tail probability, with a +1 correction followed by Holm adjustment.

MD results were summarized at trajectory and complex levels using common-pocket, other-surface, and whole-protein contact descriptors. BAEL sensitivity analyses used within-target correlations, overlaps, rank changes, and the resampling procedures specified in the Supplementary Methods.

**Manuscript preparation**. OpenAI ChatGPT (GPT-5.5) and Codex were used for language editing, manuscript organization, consistency checks, and code-assisted spreadsheet preparation. All five authors reviewed the incorporated outputs against the source data and analysis records. The authors retain responsibility for the analyses, interpretations, and manuscript.

## 5. Conclusions

This study prioritized 182 natural products across 189 candidate–target associations involving ten *Pseudomonas aeruginosa* PAO1 virulence-associated proteins. The resulting collection combines coverage of six virulence-related functions with broad scaffold diversity, offering multiple chemical starting points for target-specific investigation. BAEL largely preserved docking-led selections while making property-related trade-offs explicit near the selection cutoffs, with high selection overlap under the examined Access, Exposure, and Liability perturbations. The accompanying structural and trajectory analyses provide context for interpreting the proposed associations and organizing their experimental evaluation. The independent LasB analysis highlighted the importance of considering control-set composition and analysis design when interpreting score discrimination. Structural support varied among targets, and no prospective biochemical or cellular validation was performed; binding and antivirulence activity therefore remain unconfirmed. By linking candidate rankings to structural evidence, predicted compound properties, and proposed assay routes, this study provides a practical resource for planning the next stage of experimental antivirulence research.

## Figures and Tables

**Figure 1:**
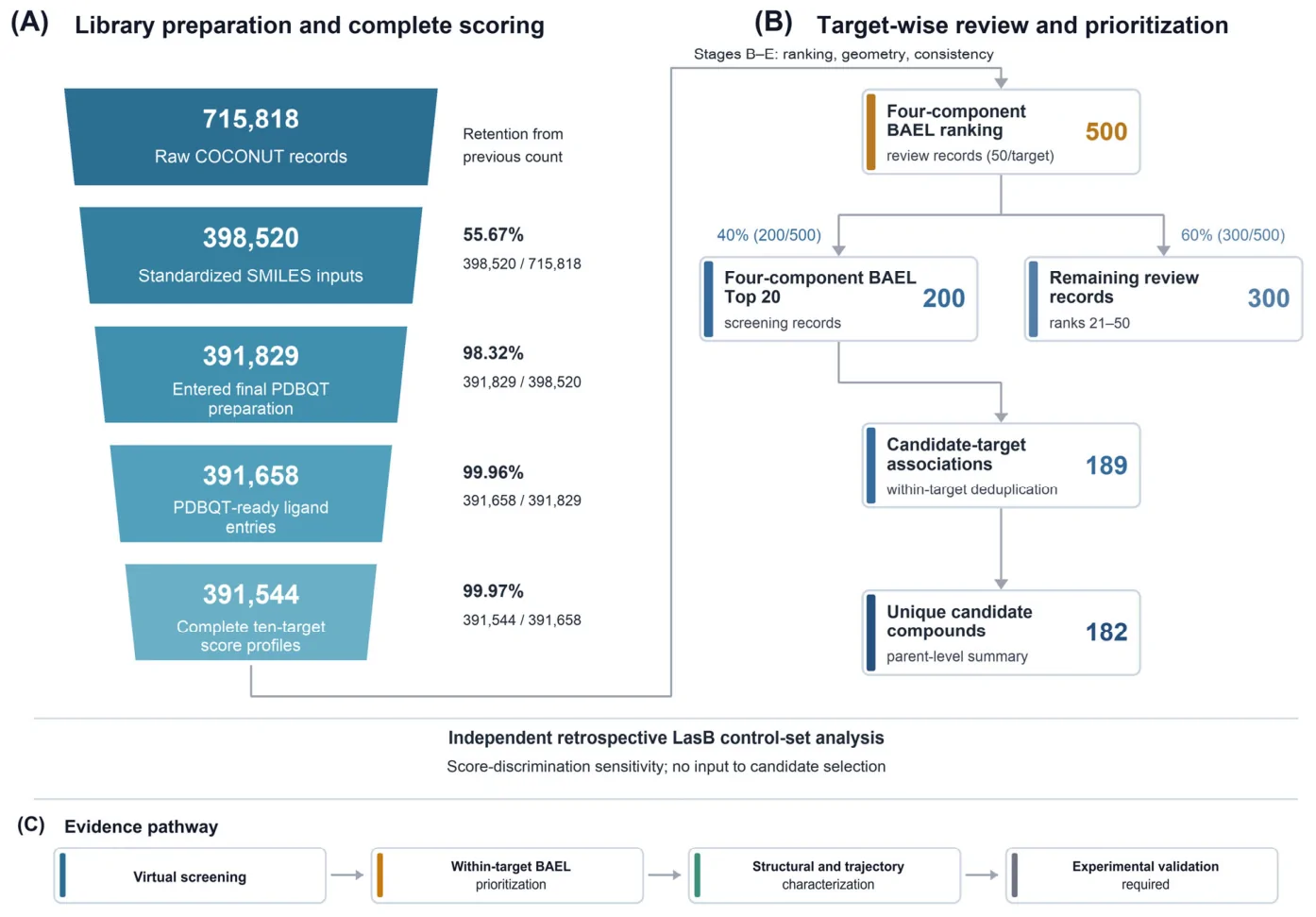
Screening workflow and candidate consolidation. (**A**) Library preparation and complete scoring across ten targets; percentages are relative to the preceding displayed count. (**B**) Within-target Binding–Access–Exposure–Liability (BAEL) prioritization of 500 review records (50 per target), retaining 200 records (40%) and leaving 300 records ranked 21–50 (60%). Consolidation yielded 189 candidate–target associations involving 182 unique compounds, while retaining cross-target associations. The connector between (**A**) and (**B**) represents Stages B–E (Table S13). (**C**) Evidence pathway from computational screening and prioritization through structural and trajectory characterization to experimental validation still required. The separate LasB band denotes an independent retrospective analysis that did not inform candidate selection.

**Figure 2:**
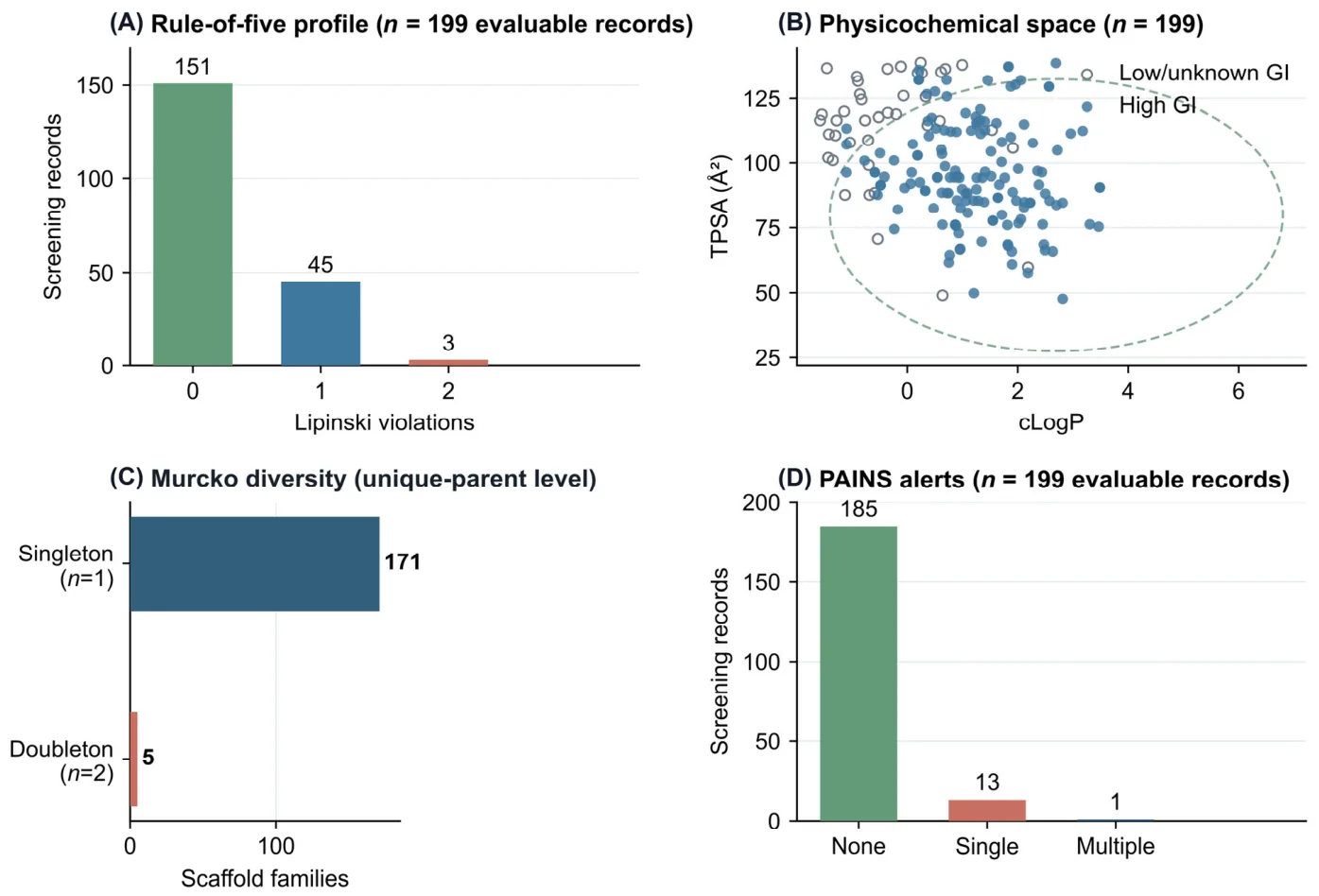
Physicochemical profiles and Bemis–Murcko scaffold diversity. (**A**) Number of Lipinski rule-of-five violations. (**B**) Calculated log octanol–water partition coefficient (cLogP) versus topological polar surface area (TPSA); filled and open symbols indicate predicted high and low/unknown gastrointestinal absorption, respectively. The dashed ellipse in (**B**) is a fixed visual guide. (**C**) Scaffold-family sizes for 181 unique compounds: 171 singleton and five doubleton families, giving 176 families in total. (**D**) Counts of PAINS alerts per record. Panels (**A**,**B**,**D**) include 199 structurally evaluable screening records. Property and alert annotations are predictions, not experimental measurements (Table S2). PAINS denotes pan-assay interference compounds.

**Figure 3:**
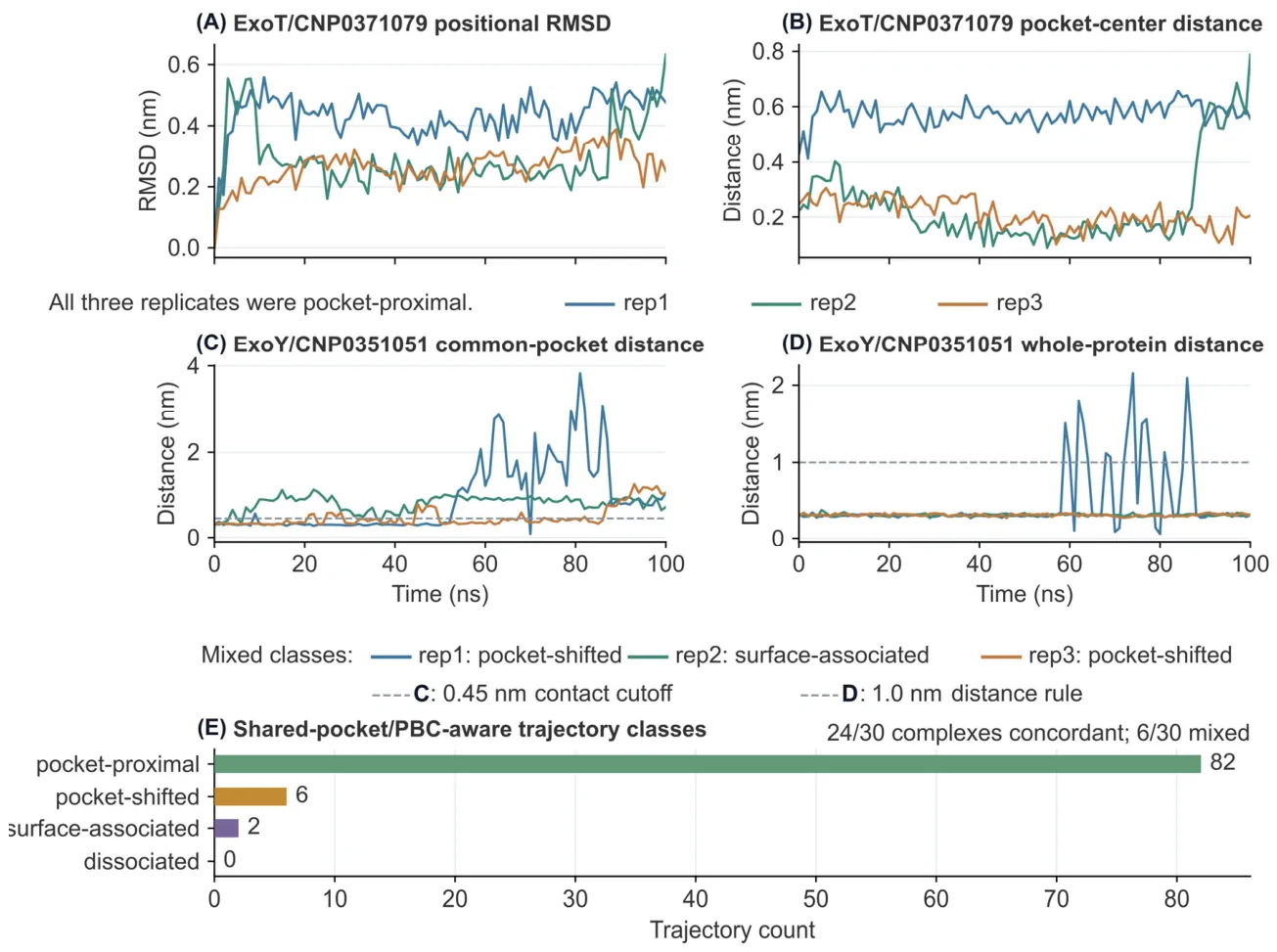
Representative molecular dynamics trajectories and geometric classifications. (**A**) Positional ligand root-mean-square deviation (RMSD) after protein-backbone fitting and (**B**) pocket-center distance for ExoT/CNP0371079. (**C**) Common-pocket minimum distance and (**D**) whole-protein minimum distance for ExoY/CNP0351051. Curves show three 100 ns replicates per complex; dashed lines in (**C**,**D**) mark 0.45 and 1.0 nm, respectively. (**E**) Classification of 90 trajectories from 30 selected complexes (three associations per target).

**Figure 4:**
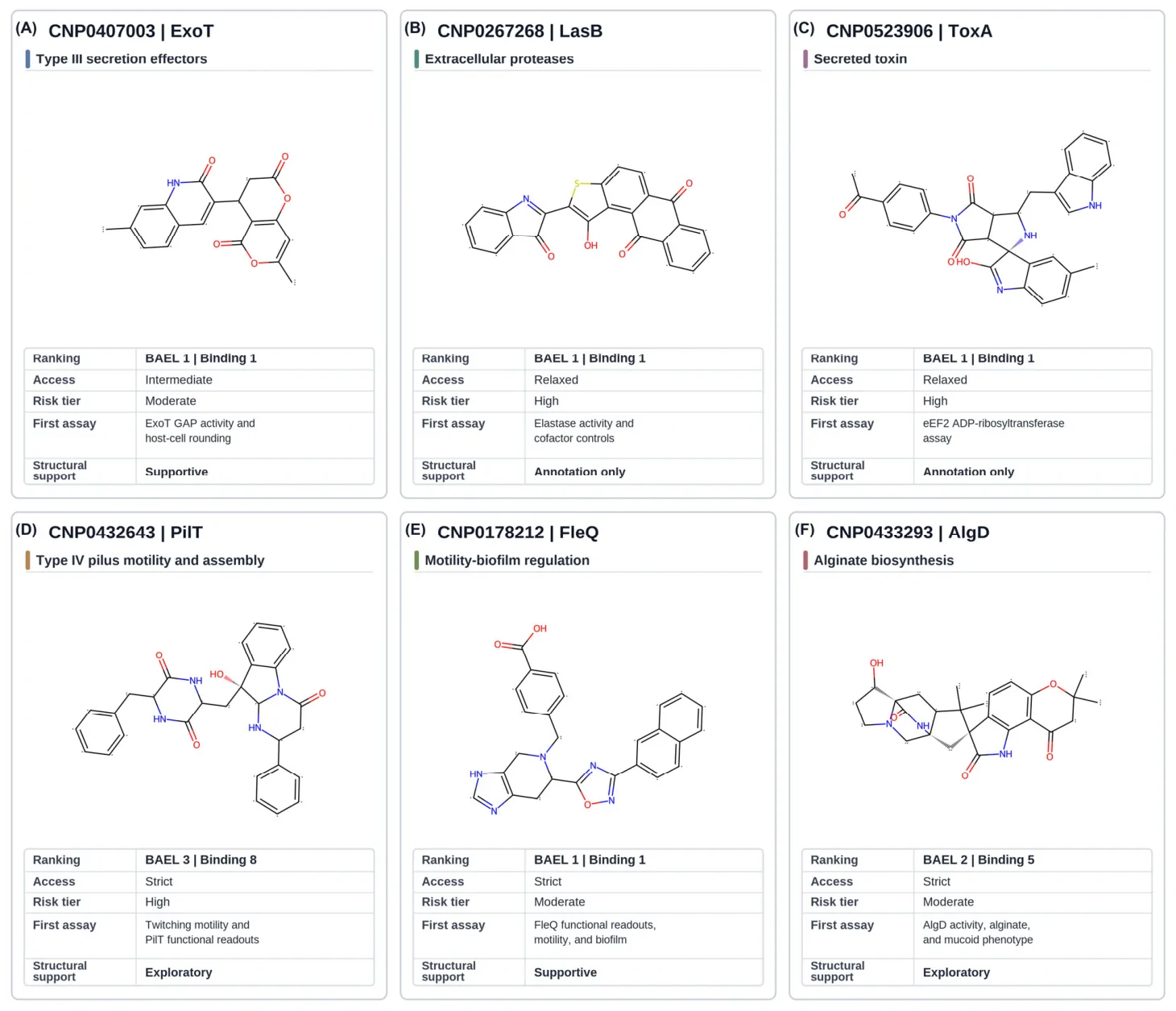
Representative candidate–target pairs and Binding–Access–Exposure–Liability (BAEL) scores across six virulence-function classes. (**A**) ExoT/CNP0407003, 0.745; (**B**) LasB/CNP0267268, 0.888; (**C**) ToxA/CNP0523906, 0.793; (**D**) PilT/CNP0432643, 0.663; (**E**) FleQ/CNP0178212, 0.690; and (**F**) AlgD/CNP0433293, 0.663. Each card shows the chemical structure, within-target BAEL and Binding-only ranks, Access class, post hoc risk tier, structural-support category, and proposed first-line assay. Scores are interpreted within targets; exact values are provided in Table S6. Risk tiers were assigned after ranking (Section 4.4).

**Figure 5:**
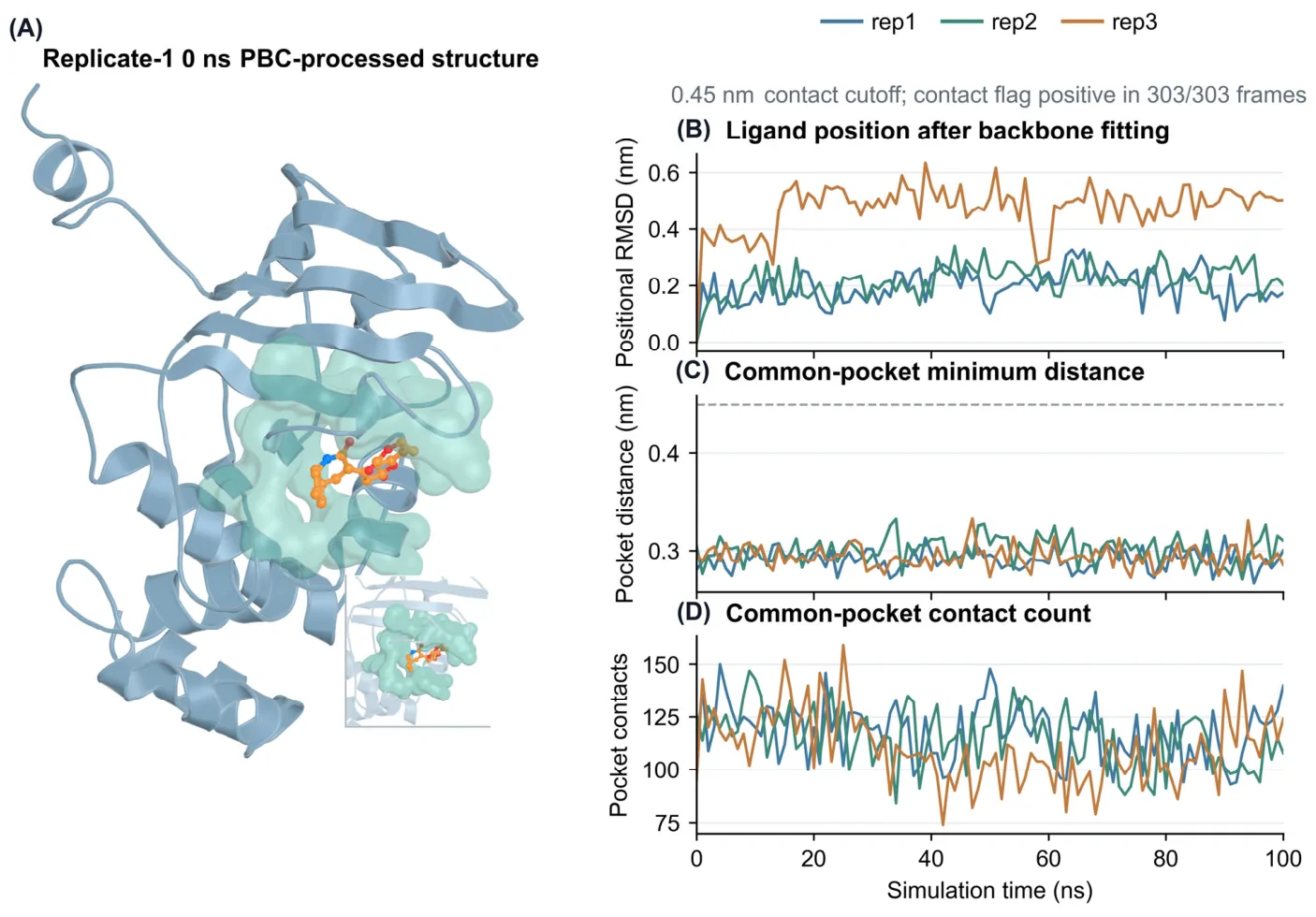
Starting configuration and trajectory descriptors for ExoT/CNP0407003. (**A**) The 0 ns configuration from replicate 1, processed for periodic boundary conditions (PBC); CNP0407003 is orange, and the shared pocket is translucent green. (**B**) Positional ligand root-mean-square deviation (RMSD) after protein-backbone fitting. (**C**) Minimum ligand–common-pocket distance. (**D**) Common-pocket contact count. Curves show three 100 ns replicates analyzed at 1 ns intervals; the dashed line in (**C**) marks the 0.45 nm contact cutoff. The Binding–Access–Exposure–Liability (BAEL) score was 0.745486, with BAEL/Binding-only ranks of 1/1. Proposed follow-up assays are listed in Table 3. These descriptors characterize pocket proximity and pose drift and do not establish binding or activity.

**Figure 6:**
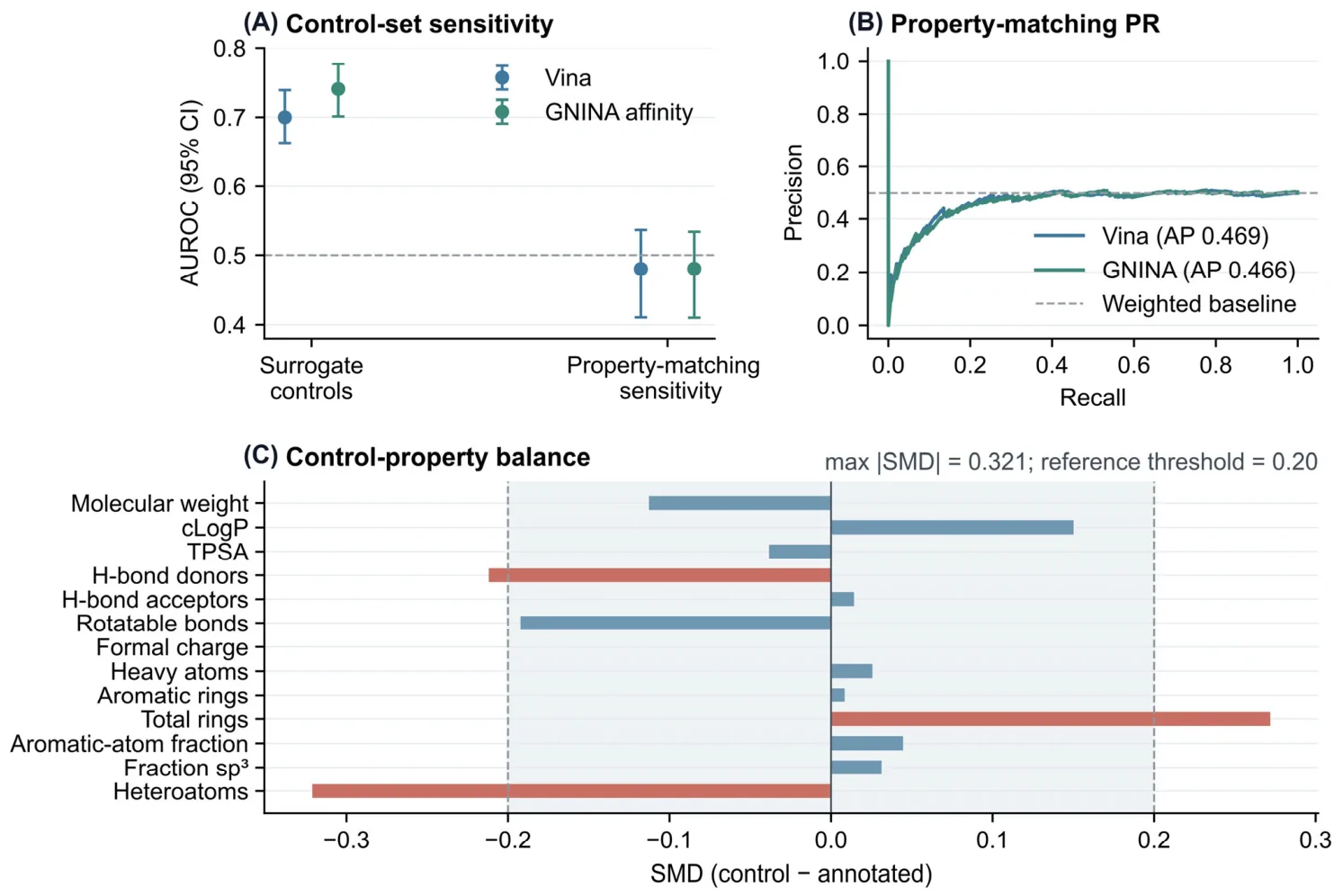
Retrospective LasB control-set sensitivity and residual property imbalance. (**A**) Areas under the receiver operating characteristic curve (AUROCs) for the Vina docking score and GNINA affinity with surrogate controls (0.700/0.740; 195 annotated parents and 893 controls) and in the attempted property-matching sensitivity set (0.480/0.480; 148 parents and 705 controls). Error bars show 95% confidence intervals. (**B**) Parent-normalized precision–recall curves for the sensitivity set; Vina/GNINA average precision (AP) values are 0.469/0.466, with a weighted baseline of 0.5. (**C**) Residual standardized mean differences (SMDs; control minus annotated); red bars indicate |SMD| > 0.20 (maximum, 0.321). Reference lines mark AUROC = 0.5, AP = 0.5, and SMD = ±0.20. Controls are unannotated comparators, and parent coverage and weighting differ between analyses.

**Table 1:** Analysis levels and retained counts.

Analysis Level	*n*	Role in the Study
PDBQT-ready library	391,658	Prepared ligand entries available for target-wise docking
Complete-profile library	391,544	Entries with Stage A Vina scores for all ten targets
Target review pool	500	50 highest-ranked Stage E records per target after score, geometry, and consistency filtering
Screening set	200	Fixed top-20 BAEL records per target from the 50-record review pools
Candidate–target associations	189	One association per target and source-parent identifier
Unique natural products	182	Distinct source-parent identifiers across all retained associations

Filtering and ranking criteria are detailed in Section 4.2 and Supplementary Table S13. Screening records are prepared ligand entries assigned to a target. Grouping the 200 records by target and source-parent identifier gives 189 associations; the highest-ranked BAEL record represents each association (Table S18). Counting source-parent identifiers across targets gives 182 compounds (Table S1), while all target-specific associations are retained. Abbreviations: BAEL, Binding–Access–Exposure–Liability; PDBQT, Protein Data Bank format with partial charges and atom types.

**Table 2:** Biological context, structural basis, and proposed first-line evaluation of the ten PAO1 targets.

Target	Biological Rationale	Access Context	Structural Basis and Scope	Proposed First-Line Assay
LasB	Extracellular proteolysis; inhibitor evidence [7,8,20,21,22]	Relaxed	Recorded 1EZM; site-based setup; Zn^2+^ and Ca^2+^ retained; no reported pose-recovery RMSD	Purified LasB elastase assay with metal-chelation and readout-interference controls
AprA	Extracellular proteolysis and immune-recognition modulation [8]	Relaxed	Prepared receptor; annotated metal site; Zn^2+^ and Ca^2+^ retained; no reported RMSD	Purified protease or culture-supernatant assay
LasA	Extracellular protease; biochemical mechanism [9]	Relaxed	Prepared receptor; TLA reference (3IT7); RMSD 0.62 Å (qualitative)	Staphylolytic or protease assay
ExoT	Host-directed RhoGAP effector [10,11,12]	Intermediate	Prepared receptor; F4W reference; RMSD 1.14 Å (qualitative)	ExoT GAP activity assay followed by assessment of host-cell rounding
ExoY	Host-directed cyclic-nucleotide production [10,11,13]	Intermediate	Prepared receptor; annotated functional site; no reported RMSD	cAMP/cGMP readout in a T3SS-compatible system
ToxA	Host eEF2 ADP-ribosylation [14]	Relaxed	Recorded 1AER; site-based apo setup; no reported RMSD	eEF2 ADP-ribosylation followed by host-cell confirmation
PilT	Type IV pilus retraction and motility [15,16]	Strict	Exploratory setup; prepared apo receptor; pose-recovery RMSD 10.78 Å	Twitching-motility assay with PilT functional readouts
PilM	Type IV pilus assembly and complex stability [17]	Strict	Exploratory setup; prepared apo receptor; pose-recovery RMSD 10.44 Å	Pilus-assembly and surface-attachment assays
FleQ	c-di-GMP-responsive motility–biofilm regulation [18]	Strict	Prepared receptor; AGS reference (5EXS); Mg^2+^ retained; RMSD 0.79 Å (qualitative)	DNA-binding or regulatory assay followed by motility and biofilm readouts
AlgD	Alginate precursor biosynthesis [19]	Strict	Exploratory apo setup; recorded 1MFZ; pose-recovery RMSD 9.35 Å	AlgD activity followed by alginate and mucoid-phenotype readouts

Pose-recovery checks provide qualitative support for ExoT, FleQ, and LasA; PilT, PilM, and AlgD remain exploratory. Table S3 provides prepared-input metadata and reference-ligand checks. Retrospective PDB correspondences and the ToxA/AlgD label differences are described in Supplementary Section S1.1. Abbreviations: RMSD, root-mean-square deviation; ADP, adenosine diphosphate; cAMP, cyclic adenosine monophosphate; cGMP, cyclic guanosine monophosphate; eEF2, eukaryotic elongation factor 2; GAP, GTPase-activating protein; T3SS, type III secretion system.

**Table 3:** Representative candidate–target hypotheses and proposed experimental evaluation.

Candidate–Target Pair	BAEL/Binding Rank	Trajectory Summary	Testing Priority and Considerations	Proposed First-Line Assay
ExoT/CNP0407003	1/1	Pocket-proximal in 3/3 trajectories	High; supportive pose recovery; intermediate access; moderate post hoc risk; no PAINS or Brenk alert	ExoT GAP activity assay followed by assessment of host-cell rounding
ExoT/CNP0371079	2/2	Pocket-proximal in 3/3 trajectories	Medium; supportive pose recovery; intermediate access; high post hoc risk; no PAINS or Brenk alert	ExoT GAP activity assay followed by assessment of host-cell rounding
FleQ/CNP0178212	1/1	Pocket-proximal in 3/3 trajectories	High; supportive pose recovery; strict access; moderate post hoc risk; permeability and efflux assessment	FleQ DNA-binding or regulatory assay followed by motility and biofilm readouts
ExoY/CNP0273911	1/1	Mixed: 2 pocket-proximal and 1 pocket-shifted trajectory	Medium; site/annotation-based setup; intermediate access; high post hoc risk; Brenk alert	cAMP/cGMP quantification in a T3SS-compatible system
LasB/CNP0267268	1/1	Pocket-proximal in 3/3 trajectories	Medium; site/metal-centered setup; relaxed access; high post hoc risk; PAINS, aggregation, metal-chelation, and readout-interference controls	Purified LasB elastase assay
ToxA/CNP0523906	1/1	Pocket-proximal in 3/3 trajectories	Medium; site/annotation-based setup; relaxed access; high post hoc risk; Brenk alert and host-cell compatibility assessment	eEF2 ADP-ribosyltransferase assay
PilT/CNP0432643 †	3/8	Pocket-proximal in 3/3 trajectories	Exploratory; non-supportive pose recovery; strict access; high post hoc risk; permeability and efflux assessment	Twitching-motility and PilT functional assays
PilM/CNP0336731 †	2/2	Pocket-proximal in 3/3 trajectories	Exploratory; non-supportive pose recovery; strict access; moderate post hoc risk; permeability and efflux assessment	Pilus-assembly and surface-attachment assays
AlgD/CNP0433293 †	2/5	Pocket-proximal in 3/3 trajectories	Exploratory; non-supportive pose recovery; strict access; moderate post hoc risk	AlgD activity followed by alginate and mucoid-phenotype readouts

Ranks are reported as BAEL/Binding-only rank. High, Medium, and Exploratory are descriptive testing-priority labels, not an additional score, activity class, or validation result. Exploratory is reserved for the three non-supportive pose-recovery setups; High and Medium distinguish cases with fewer or greater structural, trajectory, access, or post hoc risk cautions. Full BAEL components and ranks are provided in Table S6, representative Vina/GNINA and CNN fields in Table S18, absorption, distribution, metabolism, and excretion (ADME) and structural-alert annotations in Table S2, receptor evidence in Table S3, and replicate-level trajectory metrics in Table S5. Post hoc risk tiers were assigned after BAEL ranking. † Exploratory structural setup based on reference-ligand pose-recovery RMSDs of 9.35–10.78 Å.

**Table 4:** Retrospective LasB control-set sensitivity with surrogate controls and score-blind attempted property matching. Sample sizes are reported as annotated compounds/controls.

Analysis SET	Scoring Output	Weighting; *n*	AUROC (95% CI)	AP (95% CI; Baseline)
Surrogate controls	Vina docking score	Row-unweighted; 195/893	0.700 (0.662–0.739)	0.313
Surrogate controls	GNINA affinity	Row-unweighted; 195/893	0.740 (0.701–0.776)	0.372
Property-matching sensitivity set	Vina docking score	Parent-normalized; 148/705	0.480 (0.411–0.536)	0.469 (0.427–0.532; 0.5)
Property-matching sensitivity set	GNINA affinity	Parent-normalized; 148/705	0.480 (0.410–0.533)	0.466 (0.427–0.525; 0.5)

AP is the precision–recall enrichment summary. Surrogate-control AP values are point estimates; attempted-matching AP values include confidence intervals. The respective AP baselines were 0.179 and 0.5. The attempted-matching set did not meet |SMD| ≤ 0.20 (maximum, 0.321); it also differed from the surrogate comparison in parent coverage, control composition, and weighting. Controls were unannotated comparators, not confirmed LasB negatives. Abbreviations: AP, average precision; AUROC, area under the receiver operating characteristic curve; CI, confidence interval; SMD, standardized mean difference.

## Data Availability

The original contributions presented in this study are included in the article/Supplementary Material. Further inquiries can be directed to the corresponding author.

## Supplementary Materials

The following supporting information can be downloaded at: https://www.mdpi.com/article/10.3390/ph19101537/s1. Figure S1: BAEL rank sensitivity to Binding inputs, component ablation, and rule-strength changes; Figure S2: Top-20 overlap and substitutions between BAEL and Binding-only ranking; Figure S3: Within-target A/E/L permutation analysis; Figure S4: Retrospective LasB control-set analysis; Figure S5: Functional organization and Access context of the ten-target panel; Figure S6: Series-specific LasB structure–label analysis; Figure S7: Target coverage and candidates associated with two targets; Table S1: Parent-level annotations for 182 unique candidate compounds; Table S2: ADME, structural-alert, and post hoc toxicity-risk annotations for 200 screening records; Table S3: Receptor structures, docking boxes, retained ions, and pose-recovery checks for ten targets; Table S4: Bemis–Murcko scaffold families for 181 scaffold-bearing candidates; Table S5: Replicate-level MD descriptors for 90 trajectories from 30 complexes; Table S6: BAEL component scores, ranks, and top-20 membership for 500 review records; Table S7: Candidate ranks under 19 BAEL sensitivity scenarios; Table S8: Target-level BAEL decision and sensitivity summaries; Table S9: Candidate compounds associated with two targets; Table S10: Unique candidate compounds and their target assignments; Table S11: Comparison with published *P. aeruginosa* computational-screening studies; Table S12: LasB overlap and retrospective control-set summaries; Table S13: Screening-step counts and boundary scores; Table S14: Record-level data and out-of-fold predictions for the series-specific LasB structure–label analysis; Tables S15A–S15D: Model performance, bootstrap comparisons, assay transfer, and split diagnostics for the LasB structure–label analysis; Table S16: BAEL component definitions, class weights, risk rules, and sensitivity settings; Table S17: Per-complex ligand parameterization and MD simulation settings; Table S18: Candidate–target association membership and representative states; Table S19: Property-matching control set, analysis membership, and parent-normalized weights. References [88,89,90,91,92] are cited in the Supplementary Materials.

## Abbreviations

The following abbreviations are used in this manuscript:

ACPYPE	Antechamber PYthon Parser interfacE for ligand topology conversion.
ADME	Absorption, distribution, metabolism, and excretion.
ADMET	Absorption, distribution, metabolism, excretion, and toxicity.
AMBER	Assisted Model Building with Energy Refinement force field and software suite.
AUROC	Area under the receiver operating characteristic curve.
BAEL	Binding–Access–Exposure–Liability.
BBB	Blood–brain barrier.
cAMP	Cyclic adenosine monophosphate.
cGMP	Cyclic guanosine monophosphate.
CI	Confidence interval.
CNN_VS	GNINA virtual-screening ranking score; higher values indicate more favorable rankings.
CNNaffinity	GNINA neural-network affinity output; higher values indicate more favorable model predictions.
CNNscore	GNINA pose-quality score; higher values indicate more favorable pose-quality predictions.
CNP	COCONUT natural-product identifier prefix.
COCONUT	Collection of Open Natural Products database.
ECFP4	Extended-connectivity fingerprint with diameter 4.
eEF2	Eukaryotic elongation factor 2; the cellular substrate of ToxA.
ExoT	*Pseudomonas aeruginosa* T3SS effector ExoT.
FleQ	Flagellar regulator associated with motility–biofilm regulation.
GAFF2	General AMBER Force Field 2; a ligand molecular force field.
GAP	GTPase-activating protein.
GI	Gastrointestinal.
GNINA	CNN-based molecular-docking and rescoring framework.
GNINA affinity score	Affinity-oriented output used in the LasB sensitivity analysis; lower values indicate more favorable scores.
GROMACS	Groningen Machine for Chemical Simulations; molecular dynamics simulation software.
hERG	Cardiac-safety prediction endpoint.
LasB	*Pseudomonas aeruginosa* elastase B; extracellular Zn metalloprotease target.
MD	Molecular dynamics.
NPT	Constant number of particles, pressure, and temperature ensemble.
NVT	Constant number of particles, volume, and temperature ensemble.
PAINS	Pan-assay interference compounds.
PAO1	*Pseudomonas aeruginosa* PAO1, a reference strain of *P. aeruginosa*.
PBC	Periodic boundary conditions.
PDB	Protein Data Bank.
PDBQT	Docking format with partial charge (Q) and atom type (T) fields.
pkCSM	A graph-based pharmacokinetic and toxicity prediction tool.
PseudoCAP	Pseudomonas Community Annotation Project.
RDKit	Open-source cheminformatics toolkit.
RMSD	Root-mean-square deviation.
SD	Standard deviation.
SEM	Standard error of the mean.
SMD	Standardized mean difference.
SMILES	Simplified molecular-input line-entry system.
T3SS	Type III secretion system.
TIP3P	Three-point water model.
TPSA	Topological polar surface area.
VFDB	Virulence Factor Database.
