# Supplementary material for: Multi-Target Virtual Screening of Natural Products Against Virulence-Associated Proteins of Pseudomonas aeruginosa PAO1 for Antivirulence Candidate Prioritization
Source: Pharmaceuticals (Basel). 2026 Sep 28;19(10):1537. doi: 10.3390/ph19101537 (PMC13708683; doi:10.3390/ph19101537)
Supplement: Supplementary file 1 [file pharmaceuticals-19-01537-s001.zip › 03_Supplementary_All/Supplementary_Materials.pdf]

Supplementary Materials

Multi-Target Virtual Screening of Natural Products against Virulence-Associated Proteins of Pseudomonas aeruginosa PAO1 for Antivirulence Candidate Prioritization

Wenjie Chen[1], Yu Zhang[1], Jia Zeng[1], Yuan Wang[1], and Bo Cheng[1,2,*]

[1] School of Environmental Ecology and Biological Engineering, Wuhan Institute of Technology, Wuhan 430205, Hubei, China; 18792793843@163.com (Y.Z.); 15587593301@163.com (J.Z.); 18972038323@163.com (Y.W.)

2 Hubei Key Laboratory of Microbial Transformation and Regulation of Biogenic Elements in the Middle Reaches of the Yangtze River, School of Environmental Ecology and Biological Engineering, Wuhan Institute of Technology, Wuhan 430205, Hubei, China

* *Correspondence:* lt_bb@wit.edu.cn

Supplementary Methods and Results

S1.1 Production Receptors and Docking Boxes

Receptor structures, docking boxes, chain and coordinate coverage, and retained ions for all ten targets are summarized in Table S3. That table retains the recorded project labels ToxA (1AER), AlgD (1MFZ), and LasB (1EZM).

The structure-candidate records contain the PDB counterparts reported for prepared ExoT (6GNN), PilT (3JVV), FleQ (6JDI), AprA (1KAP), LasA (3IT7), ExoY (5XNW), and PilM (5EQ6). The retained coordinate-correspondence results also associate prepared ToxA with 1IKP and AlgD with 1MV8, separately from their recorded labels. These retrospective correspondences are distinct from independently documented historical source-selection records.

The F4W-associated ExoT configuration and the FleQ/AGS and LasA/TLA structures were used for ligand-centered pose-recovery checks; FleQ/AGS and LasA/TLA corresponded to PDB entries 5EXS and 3IT7, respectively. These reference-ligand structure labels are kept separate from the production-model correspondences above. Reported pose-recovery RMSDs of 2.0 Å or lower were interpreted as supportive qualitative checks, whereas values above 2.0 Å were treated as non-supportive/exploratory. PilT, PilM, and AlgD were retained as exploratory structural setups; their reported pose-recovery RMSDs were 10.78, 10.44, and 9.35 Å, respectively (Table S3). The retained inputs label their reference ligands ACP, ANP, and GDX, respectively; the source structures and source chains for these checks remain unverified. These values limit interpretation of the proposed docking geometries. AprA, ExoY, ToxA, and LasB were interpreted from site-based or annotated structural setups without a reported reference-pose RMSD.

Retained ions were Mg^{2+} for FleQ, Zn^{2+} and Ca^{2+} for AprA, Zn^{2+} for LasA, and Zn^{2+} and Ca^{2+} for LasB; PilT, PilM, AlgD, and ToxA were modeled in the apo state. Docking was performed with AutoDock Vina 1.2.6. Exact box definitions, software-build metadata, and file checksums are provided in Table S3 and the accompanying data package.

S1.2 Staged Docking and Construction of the 500-Record Review Pool

The screening workflow comprised sequential Vina docking, pose-geometry filtering, repeat-docking assessment, GNINA rescoring, and within-target BAEL prioritization. Stage A yielded complete scores for 391,544 entries across all ten targets. For each target, Stage B retained the top 3,916 records, calculated as 1% of the 391,544 complete-profile entries and rounded up to the next integer. Stage B retention at the 3,916-record boundary was determined solely by ascending Vina score. Table S13 reports target-specific boundary scores, tie multiplicities, post-filter counts, and Stage C counts.

Stage C evaluated the 250 highest-ranked Stage B records for each target. The contact metric was selected at the target-table level: contacts per ligand atom were used when density values were available; otherwise, absolute contact counts were used for all records in that table. Eligible poses were inside the docking box, had a minimum receptor distance of no more than 3.0 Å, and met a threshold of at least 0.70 contacts per ligand atom or 15 absolute contacts. Records were ordered by Vina score, minimum distance, contact metric, center distance, and ligand identifier, and up to 150 records per target entered Stage D. Stage D used three Vina runs with `exhaustiveness` = 16, `num_modes` = 10, and `energy_range` = 5 kcal/mol.

Stage E required all three Stage D runs to succeed, a score standard deviation of no more than 0.25, an inside-box pose, and complete metrics. The primary-to-mean consistency term was defined as

$$|S_{primary} - S_3|$$

and the best-to-mean term as

$$|S_{best} - S_3|$$

Smaller raw differences indicated greater consistency. Both terms were direction-aligned before within-target winsorization at the 5th and 95th percentiles and min–max scaling. The Stage E score assigned weights of 0.20 to minimum distance, the Stage C contact metric, and the three-run mean; 0.15 to score standard deviation; 0.10 to primary-to-mean consistency; and 0.05 each to center distance, the best-of-three score, and best-to-mean consistency. Ties were resolved by the best three-run score, standard deviation, minimum distance, and ligand identifier. The 50 highest-ranked eligible records under the Stage E composite ordering were retained per target; no random sampling was used.

Stage F applied five nominal seeds (11, 22, 33, 44, and 55) to all 50 Stage E records per target using `exhaustiveness` = 64, `num_modes` = 20, and `energy_range` = 5 kcal/mol. Stage F eligibility required five successful runs and a score standard deviation of no more than 0.20. The five-run mean, standard deviation, best score, Stage E score, and ligand identifier were used to define a 20-record repeat-docking support subset for each target. Stage G independently rescored the model-1 pose of all 500 Stage E records with GNINA. GNINA rescoring was considered complete when CNNscore, CNNaffinity, and CNN_VS values were available. The repeat-docking summaries served as support annotations, whereas Stage G rescoring and BAEL ranking used the complete 50-record target pools. Records were ordered by CNN_VS, CNNaffinity, CNNscore, Stage E score, and ligand identifier to obtain the GNINA ranking.

The 50 Stage E records per target formed the prespecified 500-record review pool. The four-component BAEL prioritization rule ranked all 50 records within each target and retained the top 20; ranks 21–50 remained in the review layer. The 200 screening records were grouped by target and source-parent identifier into 189 associations, each represented by its highest-ranked BAEL record (Table S18). The 182-compound summary counts source-parent identifiers across targets (Table S1); all target-specific associations are retained. No scaffold or parent-compound cap was applied. Between-target summaries are reported as coverage statistics under the fixed-quota design.

S1.3 Four-Component BAEL Prioritization Rule

S1.3.1 BAEL and the Binding Component

The prespecified, within-target BAEL prioritization rule produced a decision-support score calculated as

$$BAEL = w_B B + w_A A + w_E E + w_L L.$$

Binding (B) combined six direction-aligned channels: the Stage E pose-geometry and repeatability composite (0.35), target-normalized Vina affinity (0.25), CNNscore (0.15), target-normalized CNN_VS (0.10), one minus the target-internal min–max-scaled cnnvariance field (0.10), and Stage F repeat-docking support (0.05). The resulting B score was clipped to 0.05–1.00. More negative raw Vina and GNINA affinity values were more favorable; all normalized channels were transformed so that larger values indicated greater support.

GNINA's `cnnvariance` output functioned as an internal consistency feature after target-wise normalization. The prespecified scoring rules assigned 0.5 to designated submetrics and zero to other designated numerical inputs when required; the selected records had complete Vina and GNINA values. Repeat-docking support was 1.0 when both rank and best-of-five score were available, 0.8 when one was available, and 0.6 when neither was available.

The coefficients were prespecified operational weights. The Stage E composite received the largest weight because it integrated pocket geometry and repeat-docking consistency, whereas the separate Vina term retained the primary continuous score signal. GNINA-derived channels supplied complementary rescoring information. Because CNNscore and CNN_VS partially overlap, and Stage E also contains Vina-derived information, these coefficients specify relative influence within the operational ranking.

S1.3.2 Access, Exposure, and Liability Components

Access class and the candidate-level Access component represent different quantities. Access class is a target-level assay category (Relaxed, Intermediate, or Strict). The candidate-level Access score (A) began at 0.90, 0.80, or 0.72 according to target class and applied class-specific deductions based on molecular weight, TPSA, rotatable bonds, cLogP, and formal charge before clipping to 0.25–1.00.

Exposure (E) began at 0.88, 0.82, or 0.76 and applied class-specific deductions for cLogP, rotatable bonds, formal charge, and molecular weight before clipping to 0.20–1.00.

Liability (L) began at 1.00. Penalties were 0.15 for each severe alert, 0.07 for each moderate alert, 0.05 for each other encoded alert, 0.10 for a DROP flag accompanied by a documented exclusion reason, 0.05 for molecular weight above 750 Da, and 0.05 for TPSA above 200 Å^2. L was then clipped to 0.35–1.00. Records without an encoded alert received no alert penalty. Table S16 lists all class-specific thresholds, score directions, clipping rules, and default rules.

S1.3.3 Class Weights and Post Hoc Risk Tiers

The class-specific weights (w_B, w_A, w_E, w_L) were (0.72, 0.08, 0.05, 0.15) for Relaxed targets, (0.60, 0.15, 0.10, 0.15) for Intermediate targets, and (0.50, 0.20, 0.20, 0.10) for Strict targets. These were prespecified operational weights. All four components were available for the 500 review records. Additional GNINA, ADME, receptor, metal/cofactor, and assay-route annotations were reported separately from the four-component BAEL score. Binding received the largest weight in every class because selection remained anchored to within-target docking evidence. Access and Exposure received greater relative influence for Strict targets to represent the added membrane-permeation and efflux constraints of bacterial intracellular targets. The weights were not fitted to activity data or optimized retrospectively.

The post hoc ADME risk tier was assigned after BAEL ranking. High risk was assigned when $L < 0.70$, an encoded structural-alert token was present, or the external-toxicity consensus flag was positive. When no high-risk criterion was met, moderate risk was assigned for molecular weight above 600 Da, TPSA above 140 Å^2, Crippen cLogP above 5.0, more than ten rotatable bonds, or an external-toxicity caution. Records meeting none of these criteria were classified as low risk. Structure-dependent risk-tier analyses comprised records with RDKit-evaluable structures.

The external consensus flag combined pkCSM hERG-I with concordant pkCSM/ADMETlab calls for Ames mutagenicity, hepatotoxicity/DILI, or hERG-II/high-hERG risk. The caution flag captured non-concordant hERG-II or ADMETlab calls for Ames mutagenicity, DILI, skin sensitization, carcinogenicity, or respiratory risk. Table S16 provides the exact Boolean definitions.

S1.4 BAEL Ranking-Sensitivity Analysis

S1.4.1 Analysis Design

All sensitivity analyses used the same 500-record review pool, and the primary 200-record screening set remained fixed. Dimension-ablation scenarios compared Binding-only, B+A, B+E, and B+L rankings with four-component BAEL after renormalization of the retained weights. Additional scenarios varied the Binding input, replaced candidate-level Access with the target-class baseline, and changed A/E/L burden strengths by 25%. Outputs included within-target Spearman and Kendall correlations, rank-biased overlap, top-3/top-10/top-20 Jaccard similarity, and absolute rank changes (Table S7).

Weight sensitivity used 5,000 Dirichlet draws centered on each class-specific weight vector. Concentration parameters of 20, 50, and 100 represented progressively narrower perturbations. Candidate-level rank intervals and top-3/top-10/top-20 frequencies were summarized together with target-level correlations, top-20 overlap, and stable-core size. Random analyses used seed 20260731.

Each BAEL entrant was paired with a displaced Binding-only member by minimizing the absolute normalized B-score difference using the Hungarian algorithm. A B-score loss of no more than 0.05 defined a low-cost substitution; thresholds of 0.025, 0.075, and 0.100 were examined in sensitivity analyses. An A/E/L Pareto improvement required the entrant to be non-inferior in all three non-Binding components and superior in at least one.

For the within-target counterfactual analysis, A/E/L rows were jointly permuted 5,000 times, preserving their correlation while breaking candidate identity. One-sided empirical P values used the +1 correction and were adjusted across ten targets with the Holm method. A secondary null replaced the same number of records near the Binding-only top-20 boundary, using a 0.10 normalized B-score window when feasible. This analysis quantified the influence of candidate-specific A/E/L annotations on selection decisions.

S1.4.2 Sensitivity Results

Independent recalculation reproduced all 200 primary top-20 memberships, with a maximum numerical residual bel ow 1.0×10^{-6}. Relative to Binding-only ranking, the median top-20 Jaccard similarity was 0.818 (mean, 0.823; range, 0. 538–1.000). The rankings shared 179 of 200 memberships and differed by 21 substitutions. Nine substitutions incurred a normalized B-score loss of no more than 0.05; seven of these nine were non-inferior in A/E/L and improved at least o ne component. At thresholds of 0.025 and 0.075, six and 18 substitutions, respectively, met the low-cost definition. At t he LasB boundary, CNP0525501 (Binding rank 21; BAEL rank 19) replaced CNP0216584 (Binding rank 20; BAEL ran k 21), with a normalized B-score loss of 0.00112, no Access change, and Exposure and Liability gains of 0.10 and 0.07.

Rule-strength perturbations yielded a median full-pool Spearman correlation of 0.999 and a median top-20 Jaccard similarity of 1.000. Corresponding medians were 0.981 and 0.905 for dimension ablation and 0.653 and 0.538 for alternative Binding inputs. The choice of Binding channel therefore contributed more ranking uncertainty than the examined A/E/L perturbations.

Across eight prespecified 25% A/E/L rule-strength perturbations and ten targets, all 80 target-scenario comparisons retained top-20 Jaccard similarities of at least 0.905. For Dirichlet concentration parameters of 20, 50, and 100, median target-wise Spearman correlations were 0.994, 0.996, and 0.998; median top-20 Jaccard similarities were 0.952, 1.000, and 1.000; and median stable-core sizes were 18.5, 19, and 19 records. Across all 30 target-by-concentration summaries, the corresponding ranges were 0.968–0.999 for median Spearman correlation, 0.818–1.000 for median top-20 Jaccard similarity, and 15–20 records for stable-core size. Observed non-Binding utility exceeded the joint-permutation reference at nominal one-sided $P < 0.05$ for six targets; ExoT, FleQ, AlgD, and PilM remained significant after Holm adjustment. Because both selection and the endpoint incorporate A/E/L, these permutation results are interpreted as internal decision-influence diagnostics.

S1.5 MD Trajectory and Complex-Level Details

The MD set comprised the three highest-ranked BAEL associations per target, yielding 30 complexes and 90 trajectories. For each complex, the shared pocket was defined from the common pre-MD system as protein residues within 0.50 nm of ligand heavy atoms; the same residue set was applied to all three trajectory replicates. The residue identities, definition hashes, initial distances, and 0.80/1.55-nm core/shell radii are provided in the accompanying machine-readable shared-pocket definition table. Pocket-proximal required common-pocket or pocket-core occupancy of at least 80%. Surface-associated required common-pocket occupancy below 20% and other-surface occupancy of at least 80%. Sustained dissociation required loss of whole-protein contact, a minimum heavy-atom distance above 1.0 nm, and at least 10 ns of continuous separation on the 1-ns analysis grid; all other trajectories were pocket-shifted.

Of the 90 trajectories, 82 were pocket-proximal, six were pocket-shifted, and two were surface-associated; none met the sustained-dissociation criterion. All three trajectory replicates received the same classification for 24 complexes. Four mixed complexes had two pocket-proximal and one pocket-shifted replicate: LasA/CNP0112623, LasA/CNP0307736, ExoY/CNP0273911, and LasB/CNP0405548. AprA/CNP0376738 had two pocket-proximal and one surface-associated replicate, whereas ExoY/CNP0351051 had two pocket-shifted and one surface-associated replicate. These proportions apply only to the selected three-per-target subset.

Each complex was analyzed across three 100-ns trajectory replicates. After minimization, each system underwent 100 ps of NVT equilibration at 300 K and 500 ps of NPT equilibration at 1 bar. End-of-NPT dodecahedral box-vector lengths averaged 9.360 nm for a, b, and c (range, 6.845–11.636 nm), and the systems contained a mean of 18,771.4 TIP3P water molecules (range, 6,719–34,262).

For each complex, the three replicates shared one solvated coordinate set before NVT. NVT used gen_vel=yes and gen_seed=−1, which requested pseudo-random velocity generation, but the realized numerical gen-seed values were not retained in the inspected text records. The NVT logs recorded three distinct realized ld-seed values per complex for the stochastic V-rescale thermostat, 90 distinct values in total. NPT and production did not regenerate velocities.

Post hoc descriptive quality control parsed all 90 NVT and 90 NPT potential-energy series and all 90 production protein-backbone RMSD series. For each series, the final 20% of recorded time was the late window and the preceding 20% was the comparison window.

Across the production RMSD series, the late-window mean had a median of 0.295 nm (IQR, 0.206–0.491), the late-window SD had a median of 0.032 nm (IQR, 0.019–0.072), and the late-minus-comparison-window mean difference had a median of −0.001 nm (IQR, −0.038 to 0.024). Median relative potential-energy changes between those windows were 0.000% for NVT (IQR, −0.023% to 0.030%) and 0.000% for NPT (IQR, −0.011% to 0.018%). Per-series means, dispersion, linear slopes, window changes, source paths, and SHA-256 values are provided in Method Assets MD_descriptive_QC.tsv and MD_descriptive_QC_summary.tsv. No prespecified numerical stabilization or convergence threshold was recovered, so these post hoc summaries are descriptive and are not used to classify trajectories as formally converged.

Gasteiger charges were used for 29 complexes and AM1-BCC for ExoT/CNP0339020.0, with the same ligand parameterization applied across the three replicates of each complex. All simulated ligand topologies had net charges of approximately zero. Replicate-level trajectory descriptors and per-complex ligand-parameterization and simulation settings are provided in Tables S5 and S17.

The ExoT/CNP0407003 structure shown in the main manuscript was obtained from the PBC-processed 0-ns configuration of trajectory replicate 1. The plotted descriptors were calculated from all three trajectory replicates.

S1.6 LasB Retrospective Control-Set Analysis

The retrospective control-set analysis and the series-specific structure–label analysis used different compounds, endpoints, and evaluation designs. This section describes the control-set analysis; the series-specific analysis is reported separately in Section S1.7.

The surrogate-control analysis included 195 parent compounds with LasB assay annotations and 893 evaluable surrogate controls. Controls for the score-blind attempted property-matching sensitivity analysis were selected before any docking or GNINA score was inspected. Exclusions removed database-annotated structures, surrogate controls, main-study candidates, exact duplicates, exact annotated-parent Murcko-scaffold overlaps, and structures with an ECFP4 Tanimoto similarity of at least 0.35 to any annotated parent.

Tier A required exact formal charge and aromatic-ring count. Additional tolerances were ±1 total ring, ±15% molecular weight, ±1.0 cLogP, ±15 Å^2 TPSA, ±1 hydrogen-bond donor, ±1 hydrogen-bond acceptor, ±2 rotatable bonds, ±15% heavy-atom count, ±0.10 aromatic-atom fraction, and ±0.15 fraction sp^3. Parents with fewer than three Tier-A candidates entered sequentially relaxed tiers, in which fraction sp^3, aromatic-atom fraction, and heavy-atom tolerances were expanded to 0.25, 0.15, and 20%, respectively. Formal charge, aromatic-ring count, structural validity, collision, and similarity constraints remained fixed across tiers.

Selection was deterministic, limited to five controls per parent, and globally nonredundant at the source-entry level. At least one control was selected for 148 of 195 annotated parents (75.9%). Among the 47 uncovered parents, 46 had no Tier-A eligible candidate; one parent had one Tier-A eligible candidate but received no control after source-entry allocation. The initial allocation yielded 714 rows with 714 unique source IDs. Standardized-InChIKey deduplication then retained 710 unique structures, of which 705 had complete four-score profiles; all 148 covered parents remained represented, and each contributed the same total weight to the parent-normalized analysis. Post-selection balance diagnostics exceeded the prespecified $|SMD| \leq 0.20$ criterion for heteroatom count (−0.321), total-ring count (0.272), and hydrogen-bond donors (−0.212); the attempted-matching set was therefore not treated as fully balanced. Table S19 reports all selected controls, matching tiers, parent assignments, score-profile status, analysis membership, and weights.

For the covered-parent analysis, each of the 148 assay-annotated parents had weight 1/148. For a parent with k complete-score controls, each control had weight 1/(148 × k). The assay-annotated and control groups therefore each had total weight 1, and the weighted class-prevalence reference for average precision was 1/(1 + 1) = 0.5. The 853 primary-analysis membership and weight records are provided in Method Assets LasB_primary_analysis_weights.tsv.

Figure S4 summarizes score discrimination and property balance; Supplementary Table S12 documents the absence of overlap between the LasB annotation/control records and the prospective candidate-selection layers, and Table S19 provides attempted-matching membership and parent-normalized weights.

S1.7 Exploratory Series-Specific LasB Structure–Label Analysis

Cross-fold domain checks used exact Bemis–Murcko scaffolds [88]. Structure groups were defined with radius-2, 2,048-bit ECFP4 fingerprints and Butina clustering at a Tanimoto-similarity cutoff of 0.50 [89]. Five-fold outer validation used StratifiedGroupKFold without shuffling, with the 13 predefined structure clusters as groups and assay-by-label classes for stratification. Inner tuning used up to three grouped folds, and stochastic estimators used seed 20260730. Out-of-fold predictions were converted to fold-specific percentile ranks before pooling [90].

The dataset contained 192 experimental-source records: 160 K_i and 32 IC_{50} records [91–92]. Fixed labels comprised 65 positive-labeled and 127 negative-labeled records. Fifty-four negative-labeled K_i records contributed categorical assay labels rather than numerical potency values. The LasB-specific model used a 512-bit radius-2 ECFP4 fingerprint and seven docking-derived channels. Eighteen candidate model families were examined. Full model specifications, software versions, split definitions, and transfer results are provided in Supplementary Tables S14–S15.

The ECFP4-plus-score model combined a 512-bit radius-2 ECFP4 fingerprint with seven docking-derived channels: negative four-seed mean Vina score, negative GNINA affinity, CNNscore, CNN_VS, negative four-seed Vina standard deviation, geometry support, and Vina ligand efficiency calculated as the negative four-seed mean divided by heavy-atom count. Access, Exposure, and Liability were excluded. All seven channels were complete for the 192 records. Median imputation and standard scaling were fitted within each training pipeline. Record-level inputs and out-of-fold predictions are provided in Supplementary Table S14.

Leakage-control clusters were generated separately using radius-2, 2,048-bit ECFP4 fingerprints and Butina clustering at a Tanimoto-distance cutoff of 0.50. The five outer folds contained 66, 46, 35, 24, and 21 records. Inner validation used the smaller of three folds or the number of available structure groups. Paired uncertainty estimates used 5,000 grouped bootstrap resamples. The design therefore constitutes nested internal cross-validation.

Four-seed Vina, the independent supervised Binding baseline, ECFP4-only logistic regression, and the ECFP4-plus-score model yielded pooled out-of-fold AUROC/average-precision values of 0.499/0.397, 0.495/0.399, 0.701/0.553, and 0.694/0.571, respectively. The AUROC difference between ECFP4-plus-score and Vina was 0.195, with a descriptive selection-conditional grouped-bootstrap 95% interval of 0.022–0.464. ECFP4-plus-score and ECFP4 alone showed comparable performance (difference, −0.007; 95% CI, −0.027 to 0.041). The model was selected from 18 candidate families using the same development resource, so these intervals are interpreted as descriptive and selection-conditional.

The strict-97 subset comprised 43 K_i A_plus records and 54 K_i I_assay records with categorical assay labels rather than numerical potency values. ECFP4-plus-score and Vina yielded AUROCs of 0.656 and 0.578, respectively; the difference interval crossed zero (−0.081 to 0.315). Within-assay ECFP4-plus-score AUROCs were 0.845 for IC_{50} and 0.627 for K_i, compared with 0.684 and 0.653 for ECFP4-only. Positive prevalence differed between the IC_{50} and K_i strata (22/32, 68.8%, vs. 43/160, 26.9%), and all 13 structure clusters were assay-specific.

Bidirectional transfer was weak. Training on the IC_{50} series and evaluating on the K_i series yielded an AUROC of 0.448; the reverse direction yielded 0.173. Exact Murcko-scaffold separation defined only six highly imbalanced groups; the final grouped validation therefore used the predefined structure clusters, and some exact scaffolds occurred in more than one final fold. Together, the results indicate predominantly series-specific structure–label signal and weak transfer between assay families. This analysis was separate from the four-component BAEL ranking. Complete results are provided in Table S14, Tables S15A–S15D, and Figure S6.

S1.8 Murcko Scaffold Definition

Canonical non-isomeric Bemis–Murcko scaffold SMILES were generated at the unique-parent level. Of the 182 uni que compounds, 181 had an evaluable parent structure; CNP0558840 was handled outside the structure-dependent analy ses. The 181 scaffold-bearing compounds formed 176 families, comprising 171 singletons and five doubletons. The larg est family contained two compounds.

Supplementary Tables

- Table S1: Parent-level annotations for 182 unique candidate compounds.
- Table S2: ADME, structural-alert, and post hoc toxicity-risk annotations for 200 screening records.
- Table S3: Receptor structures, docking boxes, retained ions, and pose-recovery checks for ten targets.
- Table S4: Bemis–Murcko scaffold families for 181 scaffold-bearing candidates.
- Table S5: Replicate-level MD descriptors for 90 trajectories from 30 complexes.
- Table S6: BAEL component scores, ranks, and top-20 membership for 500 review records.
- Table S7: Candidate ranks under 19 BAEL sensitivity scenarios.
- Table S8: Target-level BAEL decision and sensitivity summaries.
- Table S9: Candidate compounds associated with two targets.
- Table S10: Unique candidate compounds and their target assignments.
- Table S11: Comparison with published *P. aeruginosa* computational-screening studies.
- Table S12: LasB overlap and retrospective control-set summaries.
- Table S13: Screening-step counts and boundary scores.
- Table S14: Record-level data and out-of-fold predictions for the series-specific LasB structure–label analysis.
- Tables S15A–S15D: Model performance, bootstrap comparisons, assay transfer, and split diagnostics for the LasB structure–label analysis.
- Table S16: BAEL component definitions, class weights, risk rules, and sensitivity settings.
- Table S17: Per-complex ligand parameterization and MD simulation settings.
- Table S18: Candidate–target association membership and representative states.
- Table S19: Property-matching control set, analysis membership, and parent-normalized weights.
- Method asset: Shared-pocket residue definitions, hashes, initial distances, and core/shell radii for the 30 MD complexes.

List of Supplementary Figures

- Figure S1: BAEL rank sensitivity to Binding inputs, component ablation, and rule-strength changes.
- Figure S2: Top-20 overlap and substitutions between BAEL and Binding-only ranking.
- Figure S3: Within-target A/E/L permutation analysis.
- Figure S4: Retrospective LasB control-set analysis.
- Figure S5: Functional organization and Access context of the ten-target panel.
- Figure S6: Series-specific LasB structure–label analysis.
- Figure S7: Target coverage and candidates associated with two targets.

Supplementary Figures

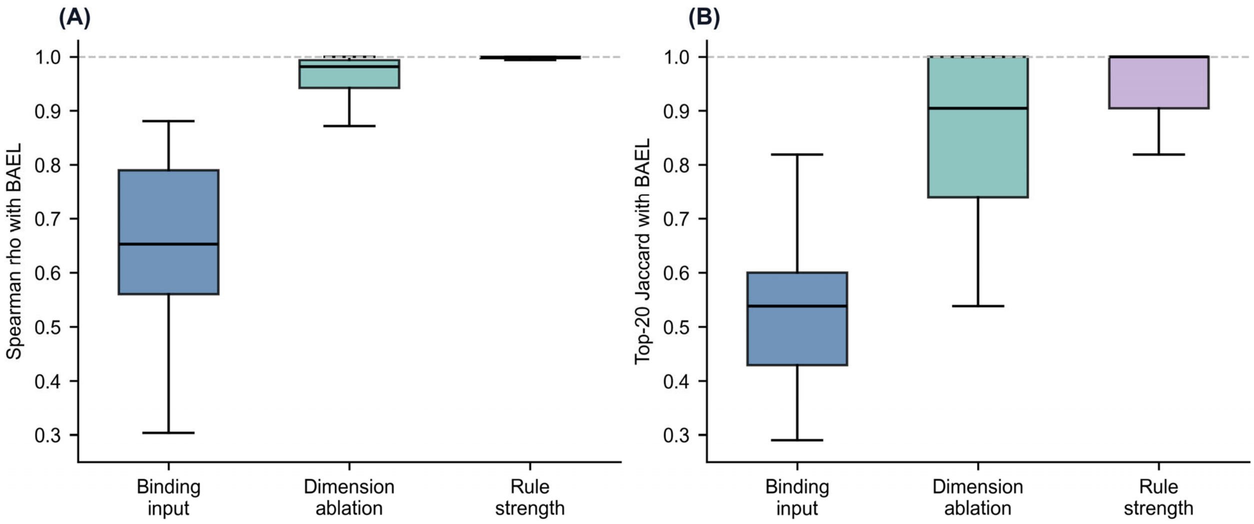

Figure S1. Sensitivity of within-target Binding–Access–Exposure–Liability (BAEL) rankings. (A) Spearman correlations with the primary BAEL ranking. (B) Top-20 Jaccard similarities. Distributions summarize scenarios involving alternative Binding inputs, component ablation, and Access/Exposure/Liability rule-strength perturbations. Higher values indicate greater agreement with the primary ranking (Supplementary Section S1.4; Tables S7–S8). Each observation is one target–scenario comparison. Boxes span the interquartile range, central lines mark medians, and whiskers reach the most extreme observations within 1.5 interquartile ranges of the box; outliers are not displayed.

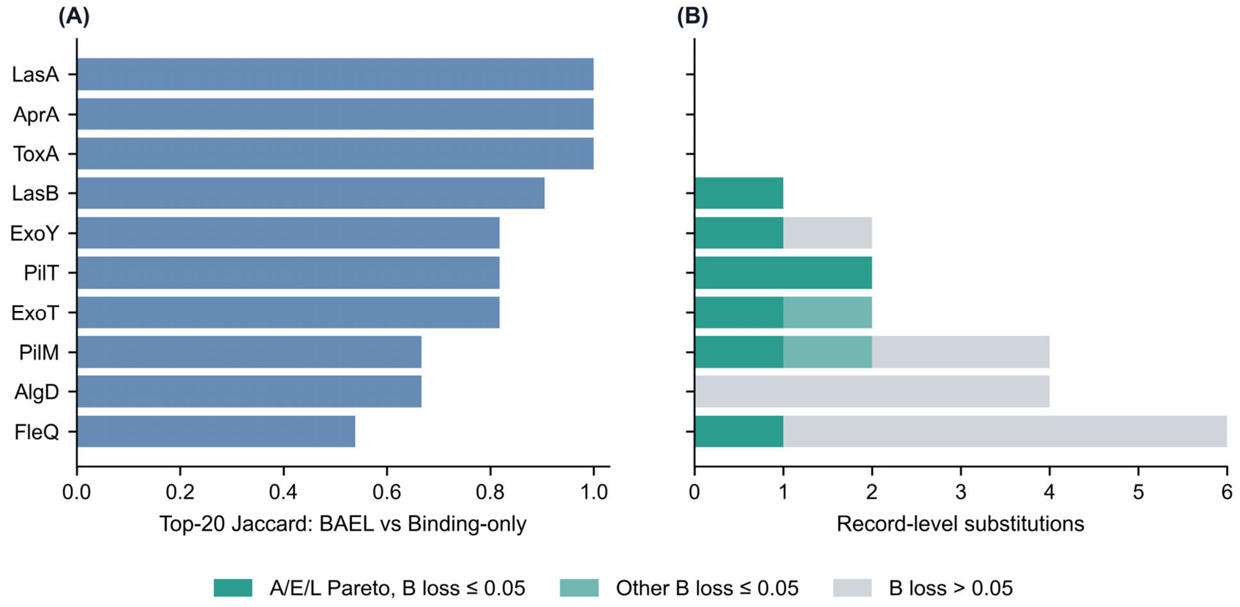

Figure S2. Selection overlap and substitutions between Binding–Access–Exposure–Liability (BAEL) and Binding-only rankings. (A) Within-target top-20 Jaccard similarities. (B) Record-level substitutions grouped as Access/Exposure/Liability Pareto improvements with normalized Binding loss ≤0.05, other substitutions with loss ≤0.05, and substitutions with loss >0.05. A Pareto improvement denotes no decrease in any non-Binding component and an increase in at least one (Supplementary Section S1.4).

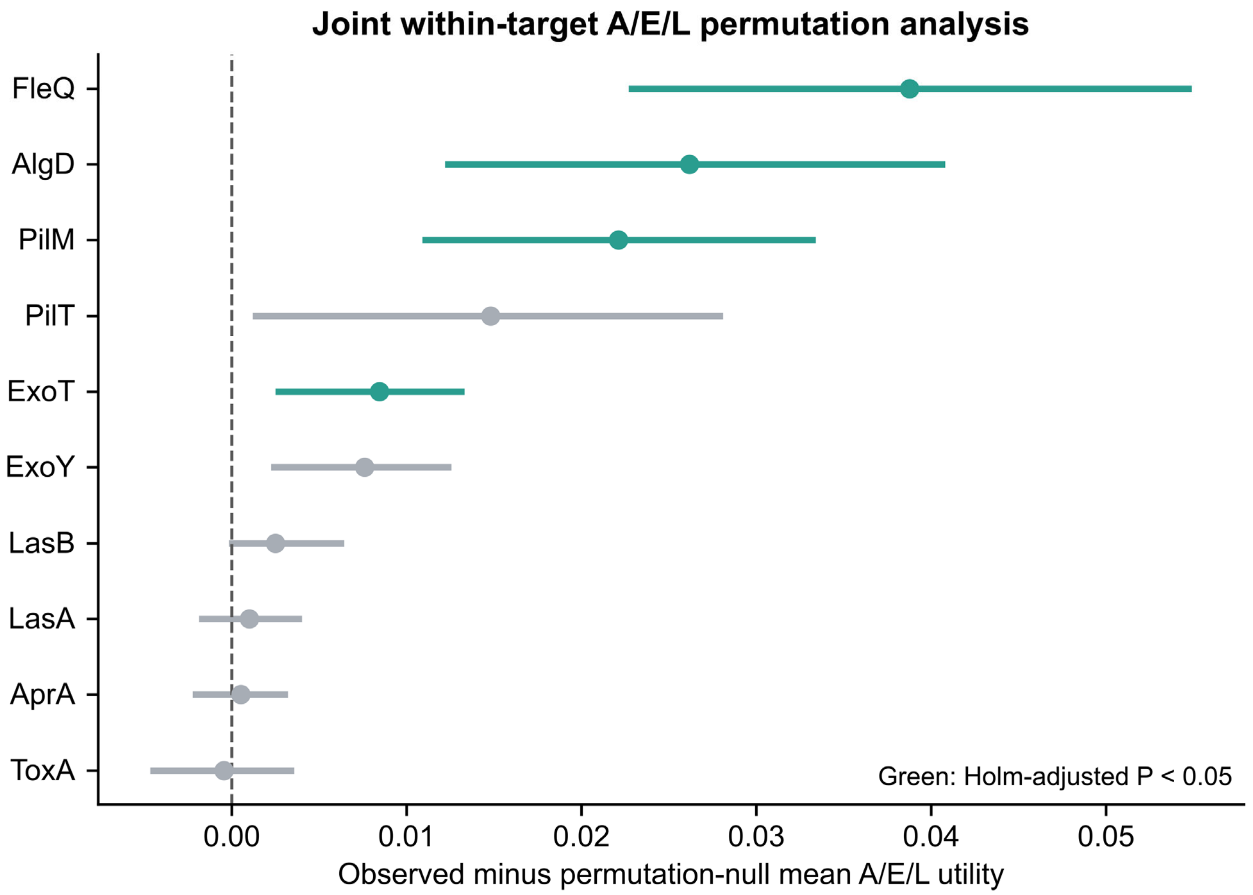

Figure S3. Within-target permutation analysis of non-Binding decision influence. Points show the observed-minus-null difference in mean Access/Exposure/Liability utility. Horizontal intervals extend from the observed utility minus the 97.5th percentile to the observed utility minus the 2.5th percentile of the permutation-null distribution. Values above zero indicate greater utility than the jointly permuted reference. Green markers denote Holm-adjusted $p < 0.05$. Because selection and the utility measure both involve these components, the results are internal decision-influence diagnostics rather than activity validation (Supplementary Section S1.4).

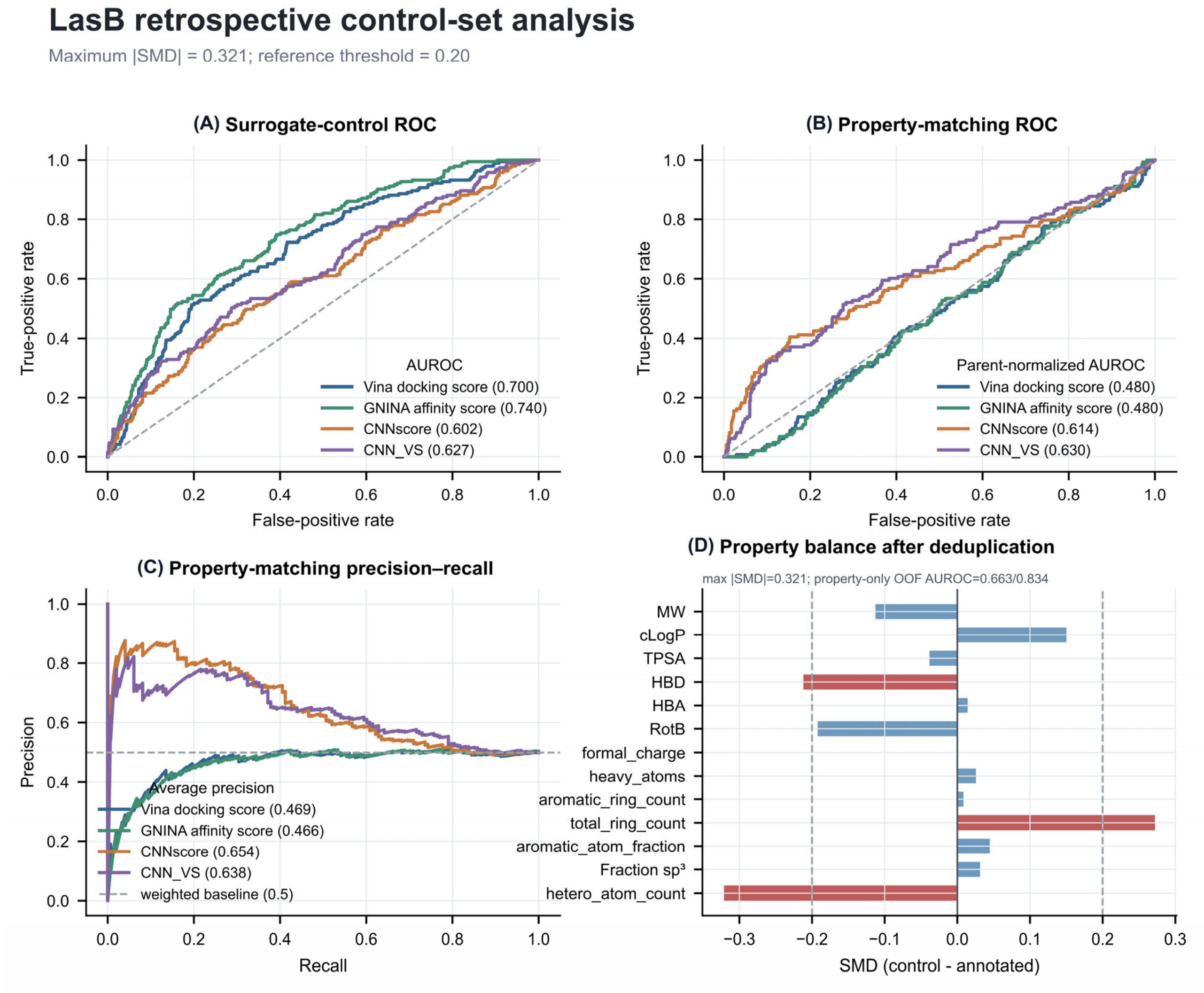

Figure S4. Retrospective LasB score discrimination and property balance. (A) Receiver operating characteristic (ROC) curves for 195 annotated parents and 893 surrogate controls. (B) Parent-normalized ROC curves for the sensitivity set of 148 parents and 705 complete-score controls. (C) Corresponding precision–recall curves, with a weighted average-precision (AP) baseline of 0.5. Panels (A–C) retain the four scoring outputs identified in the legends. (D) Residual standardized mean differences (SMDs; control minus annotated); dashed lines mark ±0.20, and the maximum |SMD| is 0.321. The sensitivity set did not meet the balance criterion. AUROC denotes the area under the ROC curve; CNNscore and CNN_VS are GNINA output fields.

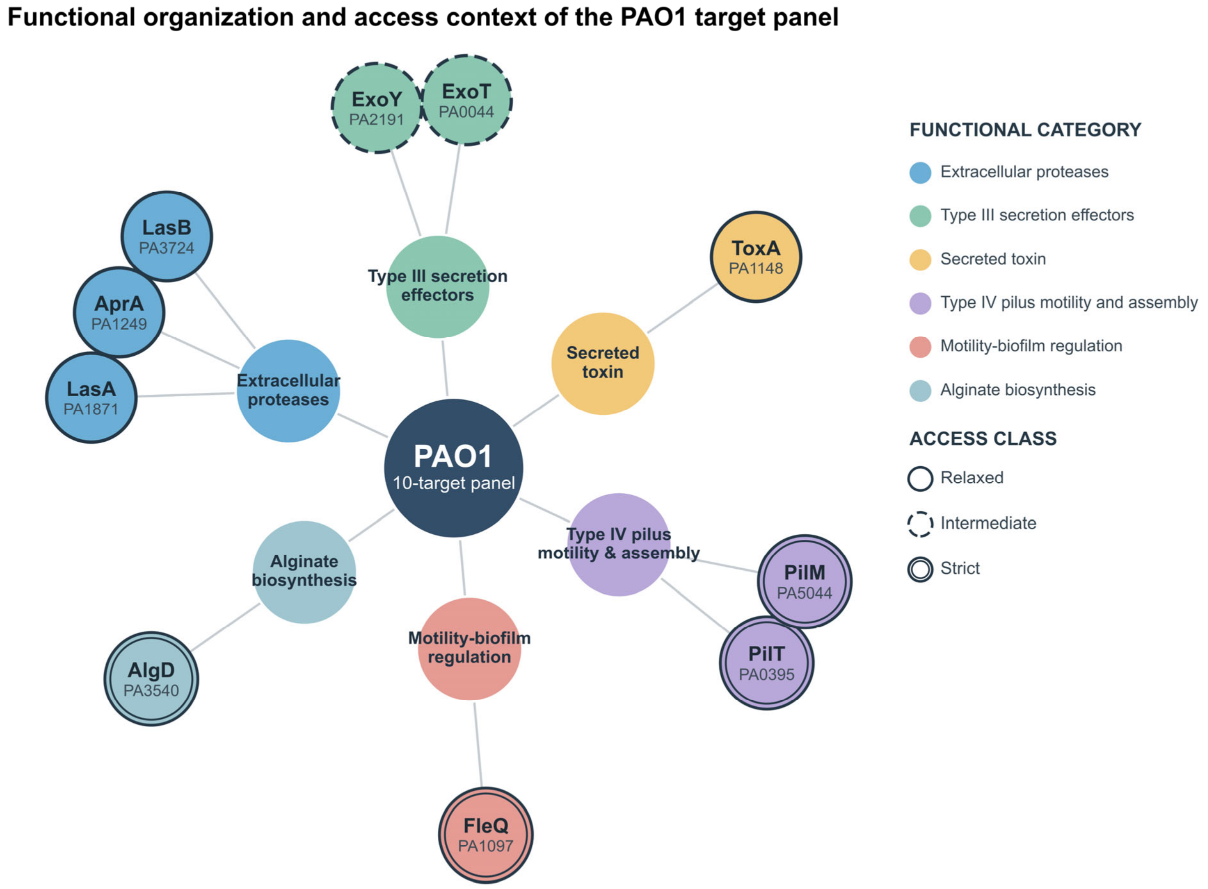

Figure S5. Functional organization and Access context of the ten-target panel. Nodes identify proteins and their locus identifiers and are grouped by virulence-related function. Functional categories and target-level Access classes follow the legend; Access classes summarize assay and exposure context.

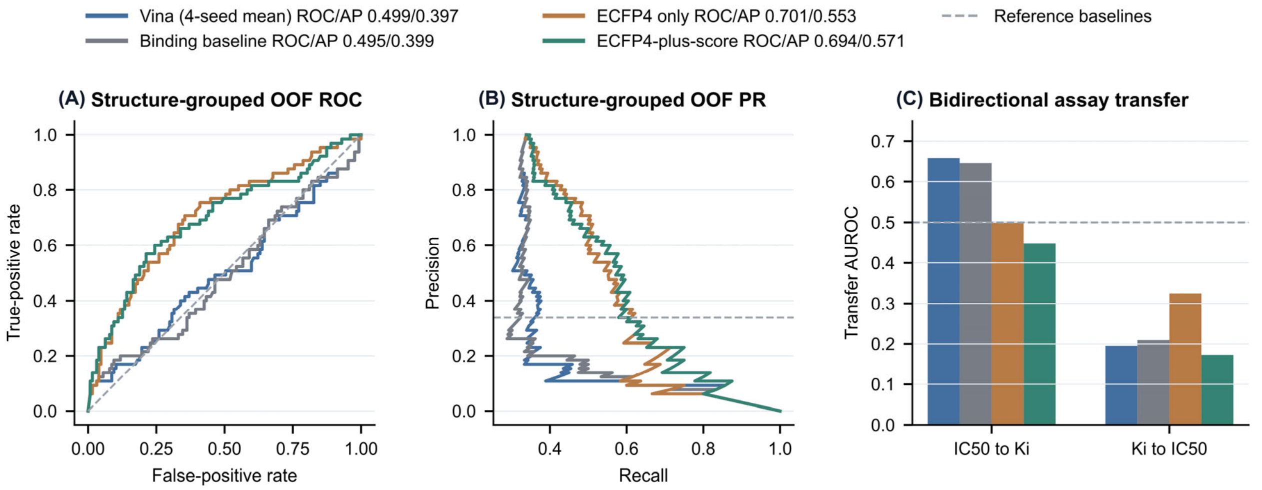

Figure S6. Series-specific LasB structure–label analysis. (A) Pooled out-of-fold receiver operating characteristic (ROC) curves. (B) Corresponding precision–recall curves. (C) Bidirectional transfer between the IC50 and Ki assay series. The four approaches are four-seed Vina, the independent supervised Binding baseline, ECFP4-only logistic regression, and the ECFP4-plus-score model. Dashed lines indicate the relevant chance or prevalence reference levels. Out-of-fold results derive from nested internal cross-validation (Tables S14–S15). ECFP4 denotes an extended-connectivity fingerprint with diameter 4; AP denotes average precision, and AUROC is the area under the ROC curve.

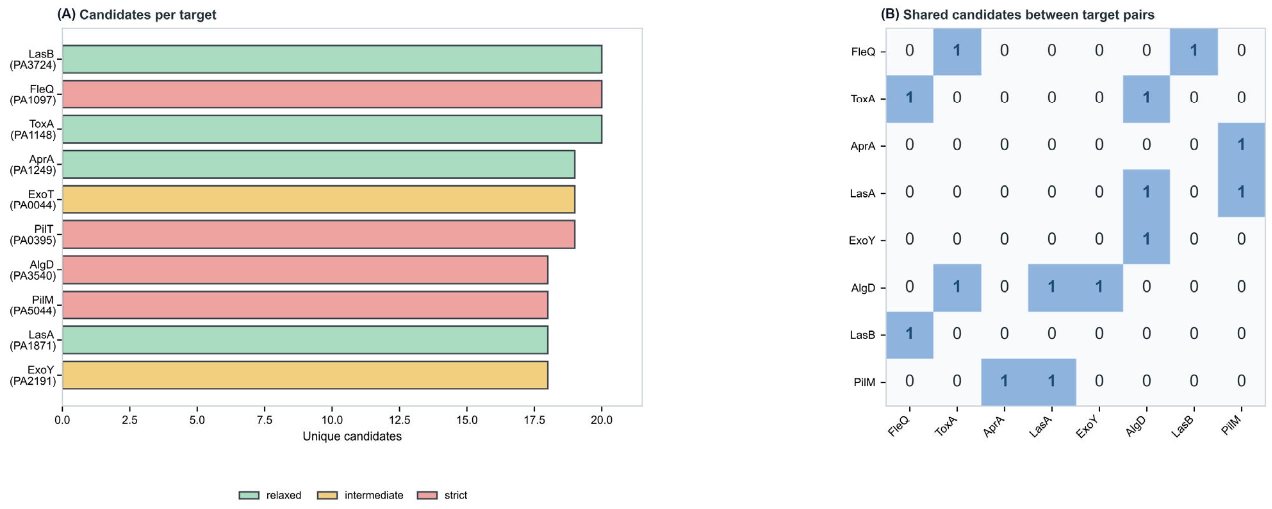

Figure S7. Target coverage and compounds associated with two targets. (A) Unique candidate counts per target after within-target consolidation. (B) Shared-compound counts for target pairs involving the seven two-target compounds. Counts describe coverage under the fixed 20-record-per-target design, not between-target enrichment.
